# Genetic Evolution and Molecular Selection of the *HE* Gene of Influenza C Virus

**DOI:** 10.3390/v11020167

**Published:** 2019-02-19

**Authors:** Wenyan Zhang, Letian Zhang, Wanting He, Xu Zhang, Baiqing Wen, Congcong Wang, Qiuhua Xu, Gairu Li, Jiyong Zhou, Michael Veit, Shuo Su

**Affiliations:** 1 MOE Joint International Research Laboratory of Animal Health and Food Safety, Engineering Laboratory of Animal Immunity of Jiangsu Province, College of Veterinary Medicine, Nanjing Agricultural University, Nanjing 210095, China; Wenyan__Zhang@163.com (W.Z.); zhangletiansky@163.com (L.Z.); vet_he@163.com (W.H.); 2017207021@njau.edu.cn (X.Z.); 15651083688@163.com (B.W.); 13661817886@163.com (C.W.); qiuhu_aki@163.com (Q.X.); 2018207022@njau.edu.cn (G.L.); 2 Key laboratory of Animal Virology of Ministry of Agriculture, Zhejiang University, Hangzhou 310058, China; jyzhou@zju.edu.cn; 3 Institute for Virology, Center for Infection Medicine, Veterinary Faculty, Free University Berlin, 14163 Berlin, Germany

**Keywords:** Influenza C virus, hemagglutinin-esterase fusion glycoprotein (HE), codon usage bias, natural selection, selection pressure

## Abstract

Influenza C virus (ICV) was first identified in humans and swine, but recently also in cattle, indicating a wider host range and potential threat to both the livestock industry and public health than was originally anticipated. The ICV hemagglutinin-esterase (HE) glycoprotein has multiple functions in the viral replication cycle and is the major determinant of antigenicity. Here, we developed a comparative approach integrating genetics, molecular selection analysis, and structural biology to identify the codon usage and adaptive evolution of ICV. We show that ICV can be classified into six lineages, consistent with previous studies. The *HE* gene has a low codon usage bias, which may facilitate ICV replication by reducing competition during evolution. Natural selection, dinucleotide composition, and mutation pressure shape the codon usage patterns of the ICV *HE* gene, with natural selection being the most important factor. Codon adaptation index (CAI) and relative codon deoptimization index (RCDI) analysis revealed that the greatest adaption of ICV was to humans, followed by cattle and swine. Additionally, similarity index (SiD) analysis revealed that swine exerted a stronger evolutionary pressure on ICV than humans, which is considered the primary reservoir. Furthermore, a similar tendency was also observed in the *M* gene. Of note, we found HE residues 176, 194, and 198 to be under positive selection, which may be the result of escape from antibody responses. Our study provides useful information on the genetic evolution of ICV from a new perspective that can help devise prevention and control strategies.

## 1. Introduction

Influenza C virus (ICV) was first identified in the United States from a human patient with upper respiratory disease in 1947 [1]. Despite its high seroprevalence rate [2,3,4], viruses were only occasionally isolated by cell culture. ICV was thought to exclusively infect humans until it was isolated from pigs [5], and recently from cattle [6]. Nevertheless, whether the bovine ICV isolates possess a zoonotic potential to cause human disease remains unknown. ICV usually causes a mild upper respiratory tract illness in children and is seldom associated with severe disease [7,8,9,10]. However, a recent study emphasized that ICV may cause a more severe disease by infecting the lower respiratory tract, and periodic outbreaks [11]. In spite of the existence of co-infection, the role of influenza C viruses cannot be ignored.

ICV is an enveloped, segmented, negative-sense RNA virus belonging to the *Orthomyxoviridae*. In contrast to Influenza A and B virus, ICV carries seven segments, with the hemagglutinin-esterase (HE) glycoprotein being the counterpart of the hemagglutinin (HA) and neuraminidase (NA) proteins of influenza A and B viruses. The HE protein is the major antigenicity determinant and plays a vital role in receptor binding, receptor destroying, and membrane fusion activities [12,13,14]. At least nine HE antigenic sites have been proposed, and in four of them, the relevant amino acid positions have been identified [15,16,17]. Moreover, in infectious virus particles, HE consists of two subunits: the HEF1 polypeptide contains the N-terminal 432 amino acids and the remaining sequence is HEF2 [18,19]. Based on phylogenetic analysis, the *HE* gene was divided into six lineages: C/Mississippi, C/Taylor, C/Aichi, C/Yamagata, C/Sao Paulo, and C/Kanagawa lineage. The diversity between ICV nucleotide sequences is low, with the *M* gene displaying the lowest and the *HE* gene the highest level of diversity. The HE glycoprotein antigenicity is highly stable, antigenic drift occurs only rarely, and thus the same antigenic types circulate over long periods of time [20].

The genetic code is redundant, allowing the modification of protein synthesis efficiency and accuracy while maintaining the amino acid sequence [21]. The codon usage pattern of viruses and hosts can provide insight into the survival, adaption, evasion from the host’s immune system, and evolution [22,23,24]. The codon usage is not random and is mainly influenced by natural selection and mutation pressure.

Until now, the factors dictating the genetic evolution of the ICV, especially in terms of codon usage bias, have not been elucidated in detail. Here, we performed a comprehensive analysis of the codon usage pattern of the ICV *HE* gene, which is based on 217 sequences obtained from 1947 to 2016. All available full-length *M* gene sequences were also included in the analysis; the limited number of whole genome sequences did not allow us to perform an in-depth full genome analysis.

## 2. Materials and Methods

### 2.1. Sequence Data

A total of 217 complete coding sequences, including four lacking stop codons (after removal of the signal peptide), of the ICV *HE* gene from 1947 to 2016 were downloaded from GenBank of the National Center for Biotechnological Information (NCBI) (https://www.ncbi.nlm.nih.gov/genbank/). Partial sequences were not included in the analysis. Furthermore, 139 *M* gene sequences were also retrieved for analysis. Among the 217 *HE* gene sequences, 213 were isolated from humans, three from swine, and one from bovine. The accession numbers and details of the ICV sequences used in this study are provided in Supplementary Materials Data S1.

### 2.2. Phylogenetic Analysis

The *HE* gene of ICV was aligned using muscle in MEGA 7 [25]. The substitution model was selected using Modelgenerator(v_851) [26] according to the Akaike Information Criterion (AIC), the Bayesian Information Criterion (BIC), and hierarchical Likelihood-ratio tests (hLRTs) values. Maximum likelihood (ML) trees were reconstructed using RAxML(v8.2.10) [27], with the GTR + GAMMA I + I model. The bootstrap value was set to 1000.

### 2.3. Selection Analysis

Sites under selection were estimated using HyPhy implemented in the Datamonkey webserver (http://www.datamonkey.org/) using four algorithms: mixed effects model of evolution (MEME), single likelihood ancestor counting (SLAC) model, fast unbiased Bayesian approximation (FUBAR), and fixed effect likelihood (FEL) [28,29,30]. The ratio of non-synonymous (dN) and synonymous substitutions (dS) was calculated to evaluate the degree of selection pressure. p values less than 0.05 for SLAC, FEL, and MEME, or a posterior probability of more than 0.9, were assumed to be significant. The sites were only considered to be under positive selection if they were supported by at least two algorithms. The structure of the HE protein (Johannesburg/1/66) was visualized using PyMOL (accession number: AAA43788). In addition, sites under positive selection were labeled on the model as red spheres.

### 2.4. Nucleotide Composition Analysis

The component parameters of the ICV *HE* gene were calculated after removing AUG, UGG, and termination codons (UAA, UGA, UAG) since AUG and UGG solely encode Met and Trp, respectively. In addition, stop codons do not encode amino acids and, thus, have no codon usage bias. The frequencies of A, U, G, C, GC, and AU were computed using BioEdit. The nucleotide frequencies of the third position of synonymous codons were calculated using the CodonW software (http://codonw.sourceforge.net/). The GC content at the first, second, and third positions (GC_1s_, GC_2s_, GC_3s_) were calculated in Emboss explorer (http://www.bioinformatics.nl/emboss-explorer/); the GC_12s_ is the mean of GC_1s_ and GC_2s_.

### 2.5. Relative Synonymous Codon Usage (RSCU)

The RSCU values of the ICV *HE* gene were calculated using MEGA 7 software to determine the codon usage patterns without the effect of amino acid composition and sequence length. The RSCU value equals the ratio between the observed usage frequency and the expected usage frequency when all the synonymous codons for one amino acid are used equally. When the RSCU value equals 1, it means there is no bias for that codon [31]. Furthermore, codons with RSCU values of more than 1.6 and less than 0.6 are considered to be over-represented and under-represented, respectively [32].

### 2.6. Effective Number of Codons (ENC)

The ENC was calculated using the CodonW software. The ENC values range from 20 (an extreme codon usage bias when only one synonymous codon is used) to 61 (no bias when all the synonymous codons are used equally for the corresponding amino acid) [33]. The ENC values were calculated as follows:

(1) $$\mathrm{ENC}=2+\frac{9}{{\bar{F}}_{2}}+\frac{1}{{\bar{F}}_{3}}+\frac{5}{{\bar{F}}_{4}}+\frac{3}{{\bar{F}}_{6}}$$

where k (k = 2, 3, 4, 6) represents k-fold degenerate amino acids, and ${\bar{F}}_{k}$ represents the mean values for F_k_, which in turn is calculated with the following formula:

(2) $$F_{k}=\frac{nS-1}{n-1}$$

where n denotes the total occurrences of the codons for the corresponding amino acid and S was calculated using the following formula:

(3) $$S=\overset{k}{\underset{i=1}{\sum }}{\left(\frac{n_{i}}{n}\right)}^{2}$$

where n_i_ is the total occurrences number of the ith codon for that amino acid.

In general, it is accepted that ENC values ≤ 35 indicate strong codon usage bias [33,34].

### 2.7. ENC Plot

ENC plots are generally used to visualize whether mutation pressure plays a vital role in codon usage bias or not. The plot is constructed by graphing GC_3s_ in the abscissa and ENC in the ordinate. If a point lies on or around the standard curve, it means that the codon usage bias is merely constrained by mutation pressure. Otherwise, other factors may also play a vital role, such as natural selection [33]. The calculations of the expected ENC values for each GC_3s_ were estimated as follows:

(4) $$\mathrm{ENC}\;\mathrm{expected}=2+s+\left(\frac{29}{s^{2}+\left(1-s^{2}\right)}\right)$$

where s stands for the value of GC_3s_.

### 2.8. Neutrality Plot Analysis

A neutrality plot graphs GC_12s_ in the ordinate and GC3 in the abscissa. The neutrality plot was produced to evaluate the degree of influence of natural selection and mutation pressure on the codon usage patterns of the ICV *HE* gene, as well as the *M* gene. When the regression coefficient is close to 1, no or weak external selection pressure exists. In contrast, if the regression curve deviates from the diagonal line, it indicates a low correlation between GC_12s_ and GC_3s_ and natural selection plays a crucial role in shaping codon usage bias [35].

### 2.9. Principal Component Analysis (PCA)

As a multivariate statistical method, PCA analysis identifies the correlations between variables and samples. PCA was based on a 59-dimensional hyperspace in accordance with the usage of the 59 synonymous codons. Codons UGG and AUG and the three termination codons were excluded from the analysis.

### 2.10. Parity Rule 2 Analysis (PR2)

The Parity rule 2 (PR2) plot graphs G_3_/(G_3_ + C_3_) in the abscissa and A_3_/(A_3_ + U_3_) in the ordinate were employed to explore the influence of mutation and natural selection on the codon usage of the ICV *HE* gene. A point for which both coordinates are 0.5 means no bias [36,37].

### 2.11. Codon Adaptation Index (CAI) Analysis

CAI analysis is performed to predict the adaptability of the ICV *HE* and *M* genes to their hosts (*H. sapiens, Sus scrofa*, and *Bos taurus*). The most common codons have the highest relative adaptability to their hosts. In addition, sequences with higher CAIs are considered to be preferred over those with lower CAIs [38]. The CAI values were computed using the CAIcal SERVER [39] (http://genomes.urv.cat/CAIcal/RCDI/) and had a range from 0 to 1. The reference datasets for *H. sapiens, Sus scrofa*, and *Bos taurus* were retrieved from the Codon Usage Database (http://www.kazusa.or.jp/codon/).

### 2.12. Relative Codon Deoptimization Index (RCDI)

The RCDI reflects the similarity of the codon usage between a given coding sequence and a reference genome. The RCDI values of different lineages of the *HE* gene were computed using the RCDI/eRCDI server [40] (http://genomes.urv.cat/CAIcal/RCDI/). The reference codon usage patterns for *Homo sapiens*, *Sus scrofa*, and *Bos Taurus* were retrieved from the Codon Usage Database. An RCDI value of 1 implies that the virus is completely adapted to its host. In contrast, RCDI values higher than 1 indicate deoptimization codon usage patterns between the virus and its host(s) [23].

### 2.13. Similarity Index (SiD)

To further reveal the effect of the codon usage patterns of the host (*H. sapiens, Sus scrofa*, and *Bos Taurus*) on the overall codon usage patterns of the *HE* gene of ICV, the similarity index was calculated as follows:

(5) $$R\left(A,B\right)=\frac{\sum_{i=1}^{59}a_{i}\;b_{i}}{\sqrt{\sum_{i=1}^{59}b_{i}{}^{2}\sum_{i=1}^{59}a_{i}{}^{2}}}$$

(6) $$D\left(A,B\right)=\frac{1-R\left(A,B\right)}{2}$$

where a_i_ is the RSCU value of a specific codon in the 59 synonymous codons of the *HE* gene of ICV, and b_i_ is the RSCU value for the same codon in the ICV host. D (A, B) represents the SiD values and it ranges from 0 to 1 [41].

### 2.14. Relative Dinucleotide Abundance Analysis

The relative abundances and CpG dinucleotide content of individual ICV *HE* and *M* genes were calculated. The odds ratio was calculated using the following formula:

(7) $$P_{XY}=\frac{f_{xy}}{f_{y}\;f_{x}}$$

where *fy* represents the frequency of nucleotide Y, f_x_ represents the frequency of nucleotide X, *f_y_f_x_* represents the expected frequency of the dinucleotide XY, and *f_xy_* is the real frequency of the dinucleotide XY. As a generally accepted criterion, when *P _XY_* > 1.23 or < 0.78, the XY pair is supposed to be over-represented or under-represented, respectively [42].

## 3. Results

### 3.1. Phylogenetic Analysis of the ICV HE Gene

**Figure 1:**
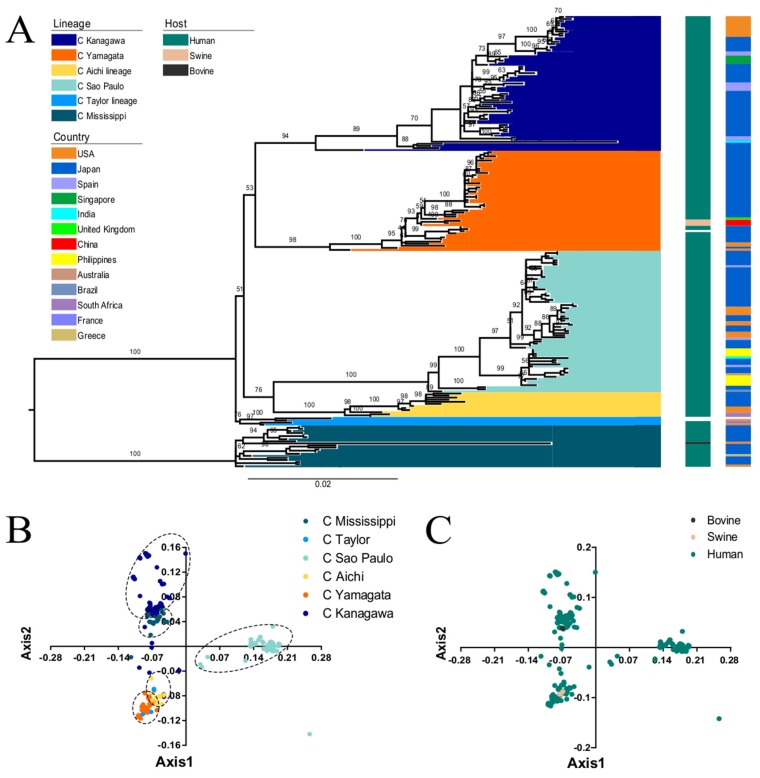
(**A**) ML phylogenetic tree of the ICV *HE* gene reconstructed in RAxML (_v8.2.10) using the GTR + GAMMA + I model and supported by 1000 bootstraps. The observed six individual lineages: C/Kanagawa, C/Yamagata, C/Aichi, C/Sao Paulo, C/Taylor, and C/Mississippi lineages are represented in dark blue, orange, light green, yellow, light blue and cyan, respectively. Coloured boxes along the right of the tree represent the hosts and countries of isolation. The host species coloured in dark green, light brown, and dark grey represent human, swine, and bovine, respectively. The individual countries are indicated with different colours. The no-coloured regions denote no related information recoded in NCBI. PCA analysis explained with the first two axes. (**B**) PCA according to the six evolutionary lineages (colours codes same as A). (**C**) PCA according to the isolated host species (colours same as A).

Phylogenetic analysis revealed that the *HE* gene could be classified into six individual lineages: C/Kanagawa, C/Yamagata, C/Aichi, C/Sao Paulo, C/Taylor, and C/Mississippi with relative high bootstrap values, which is in agreement with previous studies [43,20] (Figure 1A). Importantly, these lineages coincide with antigenic groups [44]. ICV is not only pathogenic to humans and pigs, but also to bovine (C/Bovine/Montana/12/2016), which was first identified in 2016 in the United States [45]. The ML tree showed that the C/Bovine/Montana/12/2016 strain belongs to the C/Mississippi lineage, consistent with a previous study which showed that the bovine strain is closely related to the C/Mississippi/80 lineage [45]. Additionally, the strains isolated in pigs in China clustered with the C/Yamagata lineage.

### 3.2. PCA Analysis of ICV HE Gene

PCA of the RSCU values of the *HE* gene revealed that axis1 and axis2 account for 40.65% and 18.72% of the variation, respectively (Supplementary Materials 2). Then, we plotted the axis1 against axis2 based on lineage and host to identify the specific distribution at the individual gene level (Figure 1B). In terms of lineage, there is overlap between strains of different genotypes, indicating that the ICV *HE* strains might diverge from a common ancestor [46]. C/Sao Paulo formed a separate cluster, which indicates an independent evolution. Additionally, we found overlap among the different hosts, implying similar codon usage trends (Figure 1C). It is essential to note that the analysis included only one bovine and three swine sequences. Therefore, these results require further confirmation.

### 3.3. The ICV HE Coding Regions Are Rich in A and U Nucleotides

To analyse the potential impact of compositional constraints on codon usage, the nucleotide compositions of the ICV *HE* coding sequences were calculated. The mean content (%) of A (33.48 ± 0.42) and U (26.51 ± 0.25) was higher than G (21.02 ± 0.30) and C (18.98 ± 0.18). The mean percentages of nucleotides at the third codon position of A3 (47.2 ± 1.7) and U3 (39.2 ± 0.85) were also higher than G_3_ (16.6 ± 1.67) and C_3_ (23.2 ± 0.61), indicating that A- and U-ended codons are preferred in the ICV *HE* coding sequence. The averages of GC_12s_ and GC_3s_ were 30.8 ± 0.98% and 44.6 ± 0.22%, respectively. According to the lineage classification, it is important to mention that significant differences in the mean of A_3_ and G_3_ were observed between the C/Sao Paulo lineage and the other five lineages (*p* < 0.01). Moreover, the *M* gene was also AU-rich and other nucleotide composition parameters trends were similar to the *HE* gene (Supplementary Materials Data S3).

### 3.4. Codon Usage Bias of the HE Coding Sequence

**Figure 2:**
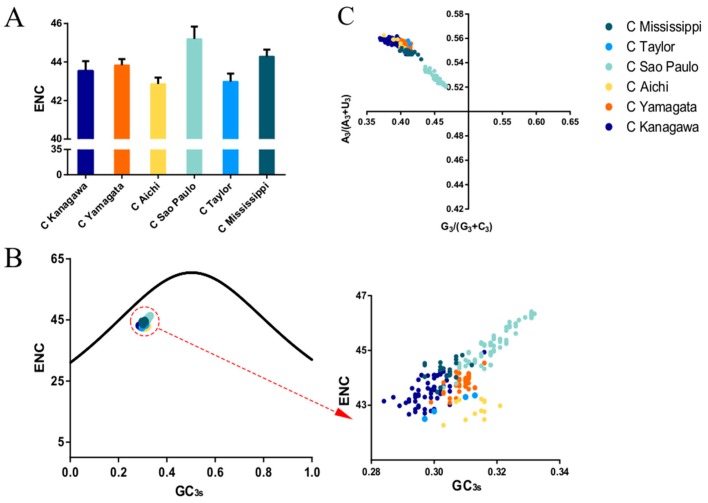
(**A**). The mean and standard deviation of effective number of codons (ENC). The respective evolutionary lineages: C/Kanagawa, C/Yamagata, C/Aichi, C/Sao Paulo, C/Taylor lineage, and C/Mississippi are represented in dark blue, orange, light green, yellow, light blue, and cyan, respectively. (**B**) ENC-plot analysis of the ICV *HE* gene. The curve represents the standard expected values. The different colour circles represent the observed ENC-GC_3s_ values of the individual lineages (colours same as A). (**C**) PR2 bias plot of the *HE* gene.

To evaluate the codon usage bias of the ICV *HE*, the ENC values were calculated. A mean value of 44.15 ± 0.92 was obtained for the overall *HE* sequences, regardless of lineages, indicating a low codon usage bias. When the lineage classification was considered, the highest ENC value (45.19 ± 0.64) was for the C/Sao Paulo lineage, and thus the lowest codon usage bias among the six lineages. In contrast, the lowest ENC value (42.86 ± 0.33) was for the C/Aichi lineage, and thus the highest codon usage bias (Figure 2A). When the structural components of the HE protein were considered, the ENC values of HEF1 and HEF2 were calculated, with a mean ENC value of 45.62 ± 0.77 and 42.42 ± 1.43, respectively. Additionally, the mean ENC value of the *M* gene was 45.19 ± 0.67.

### 3.5. The HE Gene Displays a Contrasting Codon Usage Pattern with its Hosts

RSCU analysis was performed to confirm the codon usage pattern of the ICV *HE* coding sequence. We found an extreme preference for A- and U-ended codons. Among the 18 preferred codons, 16 were A/U- ended (A-ended: 6; U-ended: 10) and two were G/C-ended (C-ended: 1; G-ended: 1). Within these preferred codons, 10 had a RSCU value > 1.6, with the highest being AGA (4.24), indicative of extreme over-presentation. We also found that the codons containing CpG dinucleotides were under-represented (RSCU < 0.6). Estimation of the overall RSCU might potentially hide lineage-specific patterns, so the RSCU values of the *HE* coding sequences were next calculated according to lineages (Table 1). There was little difference among lineages. The C/Mississippi and C/Yamagata lineages were more prone to use the UCU codon to code for Ser and the C/Taylor, C/Mississippi, and C/Yamagata lineages were more likely to use the CCA codon to code for Pro. Furthermore, we found an extreme difference between the *HE* gene and it hosts in the usage of the 18 preferred codons. The only two exceptions were the UGC codon (coding for Cysteine) used in both the ICV *HE* gene and its hosts (*H. sapiens, Sus scrofa*, and *Bos Taurus*), and AGA (coding for Arginine), which was the preferred codon for the *HE* gene in *H. sapiens*, but not in *Sus scrofa* and *Bos Taurus*. Most of these results are consistent with the RSCU analysis of the *M* gene (Supplementary Materials 2).

### 3.6. Natural Selection Dominates the Codon Usage Pattern of the ICV HE Gene

**Figure 3:**
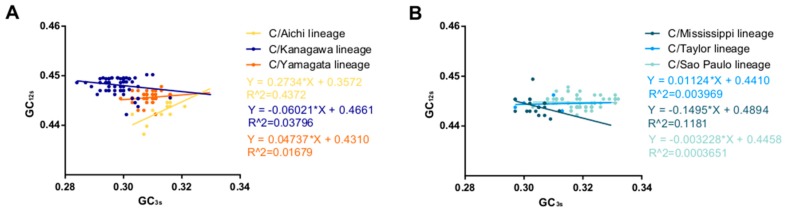
Neutrality analysis of the ICV *HE* gene. GC_3s_ are plotted against GC_12s._ (**A**) C/Aichi lineage, C/Kanagawa lineage, and C/Yamagata lineage. (**B**) C/Mississippi lineage, C/Taylor lineage, and C/Sao Paulo lineage.

**Figure 4:**
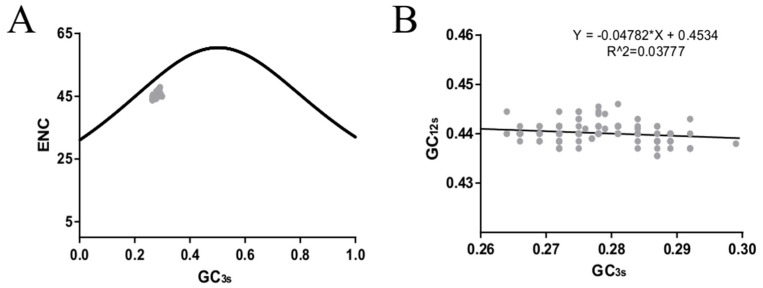
Effective number of codons (ENC) (**A**) and the neutrality analysis (**B**) of the ICV *M* gene.

ENC plots, PR2 analysis, and neutrality plot analyses were performed to explore whether the codon usage bias of the *HE* coding sequences was shaped by natural selection, mutation pressure, or both. The ENC-plot according to the different lineages showed that all the points were below the standard curves, indicating that other factors, such as natural selection, shape the codon usage of the ICV *HE* and *M* genes (Figure 2B and Figure 4A, respectively). Moreover, the PR2-plot showed that all the points were away from the origin point (0.5, 0.5) (Figure 2C), indicating a bias between the degree of mutation pressure and natural selection. Furthermore, neutrality analysis revealed the narrow distribution of GC_3s_ in all the *HE* lineages. There was only one significant correlation between GC_12s_ and GC_3s_ among the six lineages: C/Aichi lineage (R^2^ = 0.4372, *p* < 0.05). The slops of the C/Yamagata, C/Kanagawa, C/Aichi, C/Taylor, C/Sao Paulo, and C/Mississippi lineages were −0.0602, 0.0474, 0.2734, −0.00329, 0.01124, and −0.1495, respectively (Figure 3). This implies that the effect of direct mutation pressure on the codon usage bias of the six lineages is only 6.02%, 4.74%, 27.34%, 0.33%, 1.124%, and 14.9%, respectively. Natural selection plays a critical role in shaping the codon usage bias, especially in the C/Sao Paulo lineage, with the contribution of natural selection being 99.67%. As shown in Figure 4B, natural selection also plays a dominant role in shaping the codon usage patterns of the *M* gene (95.22%).

### 3.7. Dinucleotide Abundance Plays an Important Role in Shaping the Codon Usage Bias of the HE Gene

We calculated the relative abundances of the 16 dinucleotides of the ICV *HE* and *M* gene coding sequences. The occurrence of dinucleotides was found to be non-random, and CpG (mean ± SD = 0.180 ± 0.030 for *HE* gene; 0.256 ± 0.027 for *M* gene) was extremely under-represented. Interestingly, the recently reported strains (C/Bovine/Montana/12/2016) had higher CpG dinucleotide relative abundances than other ICV strains in both the *HE* (0.281) and *M* gene (0.350). However, given the limited available sequences, this needs to be further confirmed.

### 3.8. Host-Specific Codon Adaption Patterns of ICV

**Figure 5:**
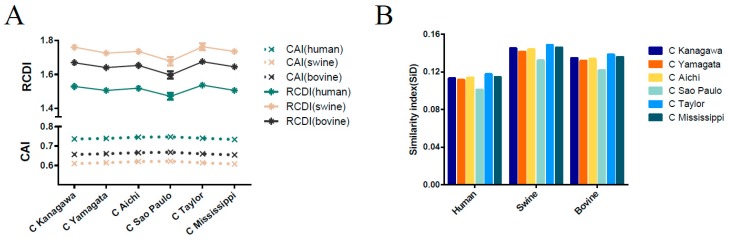
(**A**) Codon adaption index (CAI) and relative codon deoptimization index (RCDI) of the ICV *HE* gene. The abscissa denotes the evolutionary lineages and the ordinate denotes the host species. The upper panel corresponds to RCDI and the bottom panel corresponds to CAI analysis. The dark green, light brown, and dark grey indicate human, swine, and bovine, respectively. (**B**) Similarity index (SiD) analysis of the ICV *HE* gene, and the evolutionary lineages in relation to the host species.

**Figure 6:**
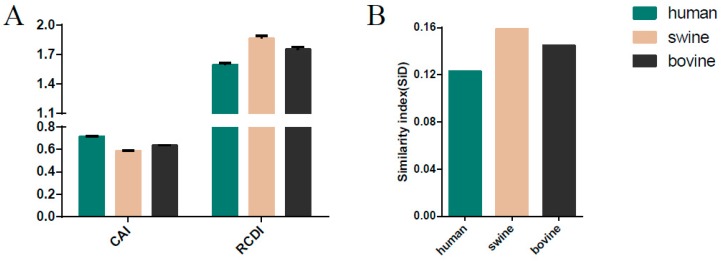
The codon adaption index (CAI), the relative codon deoptimization index (RCDI) (**A**), and similarity index (SiD) analysis of the ICV *M* gene in relation to the host species. Dark green, pink, and black indicate human, swine, and bovine, respectively.

CAI analysis was performed to determine the gene expression levels in specific hosts. We calculated the CAI values of the six phylogenetic lineages based on the three currently identified hosts. Mean CAI values of 0.74 ± 0.0056, 0.61 ± 0.0059, and 0.66 ± 0.0056 were estimated for the overall *HE* gene in relation to *H. sapiens*, *Sus scrofa*, and *Bos Taurus,* respectively. The highest CAI value was for *H. sapiens* and the lowest for *Sus scrofa*. The C/Sao Paulo lineage had the highest CAI value (0.7469 ± 0.0028 for *H. sapiens*, 0.6212 ± 0.0030 for *Sus scrofa*, 0.6674 ± 0.0027 for *Bos Taurus*) compared to the other five lineages (Figure 5A). RCDI values were calculated to compare the similarity in the codon usage of the *HE* gene and hosts. The mean of the RCDI values was highest for *Sus scrofa* (1.72 ± 0.038), followed by *Bos Taurus* (1.64 ± 0.034) and *H. sapiens* (1.50 ± 0.028), indicating the highest codon usage deoptimization for *Sus scrofa*. In terms of lineages, the C/Kanagawa and C/Sao Paulo lineages showed the highest and the lowest RCDI values in relation to the hosts (excluding the C/Taylor lineage for its limited number of sequence), respectively (Figure 5A). Moreover, the results among different ICV lineages or hosts were significant, with a p cut off < 0.001, in relation to both CAI and RCDI. For the *M* gene, the lowest CAI value and the highest RCDI value were observed in swine, similar to the *HE* gene (Figure 6A).

### 3.9. Sus scrofa Induced the Stronger Selection Pressure on ICV

SiD analysis was performed to demonstrate whether the host (*H. sapiens, Sus scrofa, Bos Taurus*) influences the codon usage patterns of the *HE* gene coding sequences (Figure 5B). The SiD value was highest in *Sus scrofa* (0.139), followed by *Bos Taurus* (0.129) and *H. sapiens* (0.108), indicating that swine exerted a stronger selection pressure than bovine and human. Additionally, the C/Kanagawa lineage displayed the highest SiD value, excluding the C/Taylor given its limited number of sequences, and the C/Sao Paulo lineage had the lowest SiD. The differences of SiD values among ICV lineages or hosts were significant with a *p* cut off < 0.001. A similar pattern was observed for the *M* gene (Figure 6B).

### 3.10. Selection Analysis of the ICV HE Gene

The relative rate of non-synonymous and synonymous mutations (dN/dS) for the ICV *HE* gene was 0.147. Site-by-site selection analysis revealed three positive amino acid sites: 176, 194, and 198 (numbering does not include the cleaved signal peptide, amino acid positions 1 to 14) under positive selection, with amino acid sites 194 and 198 supported by four algorithms and site 174 supported by three algorithms, expect for SLAC (Table 2). The site-by-site selection analysis results of the rest of the amino acid can be seen in Table S3 (Supplementary materials 2).

### 3.11. Structural Analysis of Sites under Positive Selection

To speculate what the role of the selected amino acid residues for the evolution of ICV might be, we labelled their location within the crystal structure of the ectodomain of the *HE* protein from the prototype human Johannesburg strain of ICV, which was visualized using PyMOL [47]. All three amino acids are located on the surface of the molecule in the globular head domain of the *HEF1* subunit near the receptor binding site. Residues 194 and 198 (shown as red spheres) are situated in a loop region at the very distal part of HE. This region shows amino acid substitutions between the otherwise very conserved lineages of human ICVs [48] and are part of antigenic sites A-3 and J-1 of HE of human ICV [49]. Asparagine 194 is changed to (negatively charged) glutamate in the Taylor strain of human ICV and to an (also negatively charged) aspartate residue in other human isolates. In ICV bovine isolates, residue 194 is deleted and early isolates from pigs contain a glutamate at this position, which in later isolates is reverted to glutamine. The positively charged lysine 198 is located in the same loop and is replaced by a negatively charged glutamate in the bovine HE and early porcine isolates of ICV, but exchanged by a lysine in later porcine isolates.

**Figure 7:**
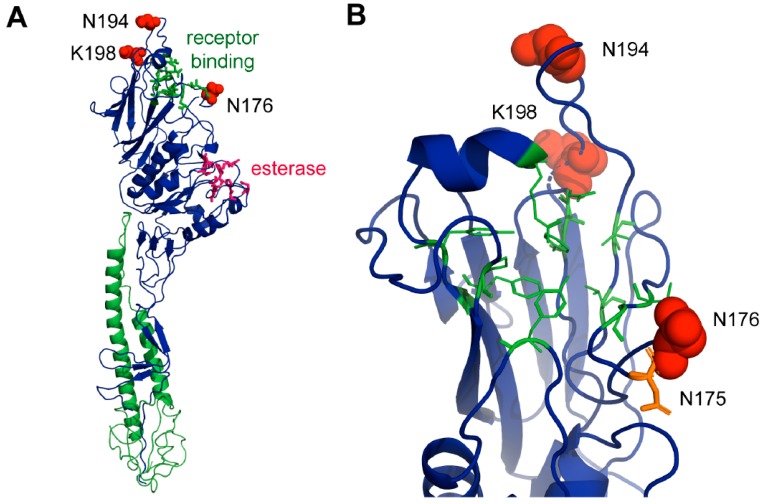
Localisation of amino acids under positive selection in the crystal structure of HE. (**A**) Complete ectodomain of an HE monomer from the 1/JHB/1/66 strain shown as a cartoon. The HE2 subunit is drawn in green and the HE1 in blue. Amino acids involved in receptor binding and esterase activity are shown as green and red sticks, respectively. Amino acids under positive selection (N176, E194 and K198) are shown as red spheres. (**B**) Magnification of the top part of HE containing the relevant amino acids. Note that N176 is part of a used N-glycosylation site (N175, N176, S177). N175 is shown as an orange stick. Figures were created with PyMol from pdb file 1FLC.

Residue 176 is located in a loop below the receptor-binding site. It contains asparagine in the HE of most isolates, but sometimes changes to an aspartate, for example, in the Miyagi clade of human ICV. Residue 176 is adjacent to the N-glycosylation site asparagine 175 (shown as an orange stick in Figure 7B). There is experimental evidence showing that this site is used, at least in the AA/50 strain that contains an asparagine at position 176 [50]. Residue 176 is part of the N-glycosylation motif (asparagine, any amino acid, serine, or threonine), in that case asparagine 175, asparagine 176, and serine 177. Since every amino acid (except a proline) is accepted by the oligosaccharyltransferase at that position, it seems unlikely that the mutation from asparagine to aspartate prevents the attachment of a carbohydrate to asparagine 175.

## 4. Discussion

ICV was first identified in humans in the United States [1] and later also in swine and cattle. ICV has a worldwide distribution, which is demonstrated by its high rate of seroprevalence, and may pose a threat to public health [4,11]. However, comprehensive studies on ICV are still limited. Recently, the *HE* genes of ICV isolates from 39 cases that tested positive from December 2014 to February 2016 in the United States were sequenced [11] and were included in our study after collapsing identical sequences. During the evolution of ICV, according to the *HE* gene, six lineages evolved, consistent with previous studies [20]. The HE sequences reported between 2014 and 2016 mainly clustered with the C/Sao Paulo and C/Kanagawa lineages, supporting a previous study that only the C/Sao Paulo and C/Kanagawa lineages have circulated since 2004. An exception is the C/Bovine/Montana/12/2016 strain, which is part of the C/Mississippi lineage [20]. The prevalence and recurrence of multiple lineages, together with cross species transmission, raises public health concerns. In this study, the codon usage patterns of the ICV *HE* and *M* genes were analysed to better understand the processes that determine ICV spread following a host range shift and to comprehend the genetic evolution process under the host pressure. This will ultimately assist the development of prevention strategies.

As previously reported, codon usage bias can be greatly influenced by the overall nucleotide composition [51]. The results of nucleotide composition showed that the ICV *HE* coding sequences were AU-rich, especially in the third position of synonymous codons. RSCU analysis also indicated that the ICV *HE* gene shows great codon usage bias towards A- and U-ended codons. It is generally accepted that directional mutation pressure explains the interspecific variation of nucleotide composition, which is primarily affected by the bias of AU/GC content [35]. Nevertheless, natural selection basically involves the selection of a specific set of codons, for instance, codons are preferred because of the host tRNAs abundance, translation selection, or the reduction of dinucleotides (CpG), which activate the host innate immunity [52,53,54,55]. As previously reported for influenza A virus, which originated from birds and has been replicating in humans for many years, the virus reduces its CpG dinucleotide frequency in its genome by directional selection [53]. Likewise, our dinucleotide analysis showed that ICV has adapted to the human host since it exhibits an extremely low CpG dinucleotide abundance in the *HE* gene and since all the codons containing CpG dinucleotides were under-represented. These results may indicate that escape from host innate immunity shapes the codon usage patterns of the ICV *HE* gene. The RSCU results also indicate that extreme differences exist in the 18 preferred codons between the *HE* gene and its known hosts. Influenza D virus (IDV), a novel genus of the *Orthomyxoviridae*, was first identified in 2011 and also contains seven genome segments [56]. Phylogenetic analysis of *PB1* gene indicated that ICV and IDV had a common ancestor [57]. However, the RSCU results of the IDV *HEF* gene showed that the codon usage patterns and the most preferred codons were very similar to its known hosts for the majority of codons [58]. It has been proposed that a consistent codon usage between viruses and their hosts allows a higher translation efficiency of the corresponding amino acids, and thus it is expected that extremely different RSCU will reduce the translation efficiency of the viral protein, but may cause it to fold properly [59]. Therefore, we hypothesized that ICV may represent a lower translation efficiency in its hosts than IDV, but may fold the viral protein properly.

In spite of the overall ENC value being higher than 35 (44.15± 0.92), the *HE* gene of ICV displayed a higher codon usage bias than many other RNA viruses, such as Zika virus [60], H1N1pdm IAV [61], H5N1 influenza virus [62], and H3N2 canine influenza virus [63]. It is hypothesized that the low codon bias of RNA viruses facilitates efficient replication by reducing the competition between the virus and the host during protein synthesis [51]. The C/Sao Paulo lineage, currently circulating, had the highest ENC values and thus the lower codon usage bias, which may favour the maintenance of successful replication. In addition, the ENC value of the HEF1 subunit (45.62 ± 0.77) is higher than that of the HEF2 subunit (42.42 ± 1.43) and the *M* gene (45.19 ± 0.67) has a higher ENC value than the *HE* gene, for reasons that remain to be further investigated. A possible explanation is that it is associated with their respective functions.

PR2 analysis and ENC-plot results revealed that natural selection plays a critical role in shaping the codon usage bias. Neutrality analysis revealed the specific magnitude of these factors in shaping codon usage bias, indicating the predominant role of natural selection in the six lineages of the ICV *HE* gene, especially in the C/Sao Paulo lineage. In order to further confirm the role of natural selection, CAI analysis was performed. CAI is frequently used to measure gene expression and assess the adaptability of viral genes to their hosts [64]. We found that the greatest adaption of ICV was to *H. sapiens*, followed by *Bos Taurus* and finally *Sus scrofa*, consistent with RCDI analysis. A low RCDI indicates good adaption to the host, while a high RCDI value implies that the virus might show a low replication rate with alternative codon usage patterns or some viral genes expressed in the latent period [40]. SiD analysis revealed that swine exerted more selection pressure on the codon usage pattern compared to humans. As previously reported, ICV may be transmitted between humans and pigs in nature [5,65]. Furthermore, the viruses isolated from swine or cattle are closely related to human ICV isolates. However, there is still no experimental evidence for interspecies transmission, and we have no data to show that ICV is a zoonotic pathogen. Whether ICV infection of swine and bovine species originated from humans and the role of bovine in the evolution of ICV remain to be further investigated. The C/Sao Paulo lineage, a current circulating lineage, revealed a greater adaptability than the other five lineages. Of note, the lowest adaption was found for the C/Kanagawa lineage, excluding the C/Taylor strain. The C/Kanagawa lineage was considered extinct because it had not been isolated since 1977. However, it re-emerged in Japan in 1996 (C/Miyagi/9/96) and acquired genes encoding internal proteins from epidemic strains of the C/Yamagata lineage [66,67,68], indicating that some difference exists in the evolution process between the C/Sao Paulo and the C/Kanagawa lineages.

Selection analysis revealed three sites under positive selection in HE: residue 176, 194, and 198. The residues under positive selection are all located in loop regions in the globular head domain of HE surrounding the receptor binding site. Since the loop containing the residues 194 and 198 is unlikely to be of structural or functional relevance for HE and since these two amino acids are located in one of the known antigenic epitopes of HE from human ICV, it seems likely that the amino acids were selected by neutralizing antibodies, i.e., virus variants with the mentioned changes could escape the antibody response of their hosts. Residue 176, which is located in a loop below the receptor binding site, is part of an N-glycosylation motif NXS/T, but the substitution at the variable X-position is unlikely to have an effect on glycosylation at asparagine 175. However, this region constitutes the antigenic site A-1 in human ICV HE [49] and thus it seems that escape from a host antibody response also selects the amino acid at this site. However, note that no significant positive selection was identified for the HE of human ICV isolates and only a few mutations affecting its antigenicity have been induced during evolution [43]. Thus, it also seems possible that the selected residues might subtly affect binding of HE to its receptor determinant 9-O-acetyl-N-acetylneuraminic acid [16,50].

In conclusion, this study showed that the codon usage bias of the ICV *HE* gene is low, but higher compared to many other RNA viruses. The major factor that has contributed to shaping codon usage is natural selection. Moreover, dinucleotides and mutation pressure also influence codon usage. Furthermore, we discovered three positive selected sites, which seem to be related to escape from the antibody responses. This is the first detailed genetic analysis of ICV devoted to extending our understanding of the mechanisms that contribute to ICV evolution.

## Figures and Tables

**Table 1:** The relative synonymous codon usage (RSCU) patterns of ICV and its hosts.

AA	Codon	Influenza C Virus *HE* Gene							Hosts		
		Overall	C/Aichi	C/Kanagawa	C/Mississippi	C Sao Paul	C/Taylor	C Yamagata	*H.sapiens*	*Sus scrofa*	*Bos taurus*
Phe	UUU(F)	**1.27**	**1.2**	**1.3**	**1.39**	**1.24**	**1.21**	**1.24**	0.93	0.79	0.85
	UUC(F)	0.73	0.8	0.7	0.61	0.76	0.79	0.76	**1.07**	**1.21**	**1.15**
Leu	UUA(L)	1.08	0.99	1.32	1.26	0.77	1	1.16	0.46	0.32	0.38
	UUG(L)	**1.87**	**1.95**	**1.66**	**1.78**	**2.26**	**1.97**	**1.62**	0.77	0.67	0.71
	CUU(L)	1.18	1.06	1.22	1.25	1.13	1.2	1.21	0.79	1.35	0.7
	CUC(L)	0.28	0.47	0.3	0.11	0.32	0.31	0.23	1.17	1.35	1.26
	CUA(L)	0.72	0.76	0.8	0.9	0.61	0.66	0.66	0.43	0.33	0.36
	CUG(L)	0.86	0.76	0.69	0.69	0.91	0.87	1.11	**2.37**	**2.68**	**2.59**
Ile	AUU(I)	**1.33**	**1.37**	**1.34**	**1.32**	**1.39**	**1.33**	**1.22**	1.08	0.91	0.98
	AUC(I)	0.86	0.78	0.9	0.85	0.83	0.85	0.89	**1.41**	**1.67**	**1.57**
	AUA(I)	0.81	0.86	0.76	0.83	0.78	0.82	0.88	0.51	0.42	0.45
Val	GUU(V)	**1.39**	**1.23**	**1.4**	**1.35**	**1.47**	**1.47**	**1.31**	0.73	0.57	0.64
	GUC(V)	0.66	0.77	0.73	0.68	0.49	0.59	0.77	0.95	1.07	1.01
	GUA(V)	0.79	0.6	0.79	0.91	0.8	0.81	0.77	0.47	0.34	0.4
	GUG(V)	1.16	1.4	1.08	1.06	1.25	1.13	1.15	**1.85**	**2.03**	**1.95**
Ser	UCU(S)	1.7	1.55	1.65	**1.71**	1.73	1.66	**1.75**	1.13	0.99	1.04
	UCC(S)	0.28	0.44	0.32	0.27	0.24	0.32	0.26	1.31	1.5	1.37
	UCA(S)	**1.74**	**1.8**	**1.72**	1.57	**1.82**	**1.82**	1.69	0.9	0.73	0.79
	UCG(S)	0.05	0	0.15	0.11	0	0	0	0.33	0.39	0.39
	AGU(S)	0.96	1.02	0.8	0.8	1.16	0.95	0.92	0.9	0.77	0.87
	AGC(S)	1.27	1.19	1.36	1.54	1.05	1.26	1.38	**1.44**	**1.62**	**1.53**
Pro	CCU(P)	**1.68**	**1.9**	**1.68**	1.35	**1.83**	1.59	1.56	1.15	1.05	1.08
	CCC(P)	0.66	0.42	0.64	0.78	0.62	0.72	0.77	**1.29**	**1.46**	**1.39**
	CCA(P)	1.59	1.68	**1.68**	**1.61**	1.42	**1.69**	**1.67**	1.11	0.94	1
	CCG(P)	0.07	0	0	0.25	0.14	0	0.01	0.45	0.56	0.53
Thr	ACU(T)	**1.75**	**1.61**	**1.79**	**1.85**	**1.71**	**1.62**	**1.77**	0.99	0.83	0.89
	ACC(T)	0.99	1.08	0.97	0.9	1.08	0.98	0.93	**1.42**	**1.68**	**1.55**
	ACA(T)	1.2	1.19	1.24	1.13	1.21	1.21	1.18	1.14	0.92	1.01
	ACG(T)	0.05	0.12	0.01	0.12	0	0.19	0.12	0.46	0.57	0.56
Ala	GCU(A)	**1.94**	**2.02**	**1.9**	**1.84**	**2.02**	**1.92**	**1.89**	1.06	0.96	1
	GCC(A)	0.54	0.61	0.51	0.54	0.5	0.56	0.6	**1.6**	**1.8**	**1.71**
	GCA(A)	1.32	1.38	1.4	1.51	1.21	1.36	1.28	0.91	0.74	0.8
	GCG(A)	0.2	0	0.19	0.11	0.26	0.17	0.22	0.42	0.5	0.48
Tyr	UAU(Y)	**1.13**	**1.1**	**1.18**	**1.23**	**1.1**	**1.11**	**1.09**	0.89	0.73	0.79
	UAC(Y)	0.87	0.9	0.82	0.77	0.9	0.89	0.91	**1.11**	**1.27**	**1.21**
His	CAU(H)	**1.61**	**1.5**	**1.73**	**1.74**	**1.57**	**1.57**	**1.46**	0.84	0.7	0.75
	CAC(H)	0.39	0.5	0.27	0.26	0.43	0.43	0.54	**1.16**	**1.3**	**1.25**
Gln	CAA(Q)	**1.74**	**1.82**	**1.79**	**1.73**	**1.68**	**1.84**	**1.73**	0.53	0.44	0.46
	CAG(Q)	0.26	0.18	0.21	0.27	0.32	0.16	0.27	**1.47**	**1.56**	**1.54**
Asn	AAU(N)	**1.35**	**1.4**	**1.3**	**1.36**	**1.37**	**1.37**	**1.37**	1.08	0.79	0.81
	AAC(N)	0.65	0.6	0.7	0.64	0.63	0.63	0.63	**1.41**	**1.21**	**1.19**
Lys	AAA(K)	**1.55**	**1.51**	**1.5**	**1.52**	**1.63**	**1.48**	**1.53**	0.87	0.76	0.78
	AAG(K)	0.45	0.49	0.5	0.48	0.37	0.52	0.47	**1.13**	**1.24**	**1.22**
Asp	GAU(D)	**1.12**	**1.04**	**1.12**	**1.01**	**1.11**	**1.14**	**1.19**	0.93	0.8	0.84
	GAC(D)	0.88	0.96	0.88	0.99	0.89	0.86	0.81	**1.07**	**1.2**	**1.16**
Glu	GAA(E)	**1.69**	**1.72**	**1.79**	**1.67**	**1.57**	**1.75**	**1.73**	0.84	0.72	0.78
	GAG(E)	0.31	0.28	0.21	0.33	0.43	0.25	0.27	**1.16**	**1.28**	**1.22**
Cys	UGU(C)	0.79	0.61	0.77	0.86	0.84	0.74	0.75	0.91	0.79	0.85
	UGC(C)	**1.21**	**1.39**	**1.23**	**1.14**	**1.16**	**1.26**	**1.25**	**1.09**	**1.21**	**1.15**
Arg	CGU(R)	0.17	0.25	0.23	0.24	0.01	0.25	0.25	0.48	0.44	0.49
	CGC(R)	0.07	0	0	0	0.24	0	0	1.1	**1.31**	1.17
	CGA(R)	0.19	0	0.31	0.23	0.25	0	0.01	0.65	0.6	0.68
	CGG(R)	0	0	0	0	0	0	0	1.21	1.29	**1.32**
	AGA(R)	**4.24**	**4.7**	**4.25**	**4.46**	**3.66**	**4.76**	**4.8**	**1.29**	1.12	1.14
	AGG(R)	1.32	1.05	1.22	1.06	1.84	0.99	0.94	1.27	1.23	1.2
Gly	GGU(G)	0.3	0.29	0.29	0.39	0.3	0.3	0.29	0.65	0.57	0.64
	GGC(G)	0.54	0.57	0.58	0.58	0.51	0.48	0.5	**1.35**	**1.46**	**1.43**
	GGA(G)	**2.51**	**2.48**	**2.62**	**2.39**	**2.42**	**2.63**	**2.52**	1	0.91	0.95
	GGG(G)	0.65	0.67	0.51	0.64	0.76	0.59	0.68	1	1.05	0.99

Preferred codons of overall and lineages of the ICV *HE* gene and potential hosts are shown in bold.

**Table 2:** Sites under positive selection.

Gene	AA	FEL		SLAC		FUBAR		MEME	
		dN-dS	*p*-Value	dN-dS	*p*-Value	dN-dS	Post.Pro	ω ^+^	*p*-Value
*HE*	**176**	**2.019**	**0.019**	5.44	0.101	**4.873**	**0.983**	**>100**	**0.03**
	**194**	**4.011**	**0.008**	**11.1**	**0.016**	**9.813**	**0.998**	**>100**	**0.00**
	**198**	**3.679**	**0.020**	**8.64**	**0.022**	**8.031**	**0.992**	**>100**	**0.03**

*p* values < 0.05 or posterior probability > 0.9 are shown in bold, ω^+^ means the value of dN^+^/dS.

## Supplementary Materials

The following are available online at http://www.mdpi.com/1999-4915/11/2/167/s1, Table S1: Explanation of the variation of ICV *HE* gene by axis, Table S2: The relative synonymous codon usage (RSCU) patterns of ICV *M* gene and its hosts, Table S3: The site-by-site selection analysis results of the rest of amino acids. Data S1: Sequence data of influenza C viruses *HE* gene and *M* gene used in this study. Data S3: The nucleotide compositions analyses of *HE* and *M* gene used in this study.
