# Supplementary material for: Genetic Evolution and Molecular Selection of the HE Gene of Influenza C Virus
Source: Viruses. 2019 Feb 19;11(2):167. doi: 10.3390/v11020167 (PMC6409753; doi:10.3390/v11020167)
Supplement: Supplementary file 1 [file viruses-11-00167-s001.zip › Supplementary materials 2.pdf]

Supplementary Materials 2

Table S1. Explanation of the variation by axis (see also eigen.coa)

Num.	Eigenval.	R.Iner.	R.Sum	Num.	Eigenval.	R.Iner.	R.Sum
01	+1.2362E-002	+0.4065	+0.4065	02	+5.6931E-003	+0.1872	+0.5937
03	+4.1631E-003	+0.1369	+0.7306	04	+2.3873E-003	+0.0785	+0.8091
05	+1.4155E-003	+0.0465	+0.8557	06	+6.0370E-004	+0.0199	+0.8755
07	+5.4706E-004	+0.0180	+0.8935	08	+4.6315E-004	+0.0152	+0.9087
09	+3.5879E-004	+0.0118	+0.9205	10	+3.1320E-004	+0.0103	+0.9308
11	+2.7100E-004	+0.0089	+0.9397	12	+1.9725E-004	+0.0065	+0.9462
13	+1.8773E-004	+0.0062	+0.9524	14	+1.7689E-004	+0.0058	+0.9582
15	+1.3922E-004	+0.0046	+0.9628	16	+1.2029E-004	+0.0040	+0.9667
17	+1.1531E-004	+0.0038	+0.9705	18	+1.0075E-004	+0.0033	+0.9738
19	+9.2863E-005	+0.0031	+0.9769	20	+8.4135E-005	+0.0028	+0.9797
21	+6.9113E-005	+0.0023	+0.9819	22	+6.1741E-005	+0.0020	+0.9840
23	+5.8198E-005	+0.0019	+0.9859	24	+5.4864E-005	+0.0018	+0.9877
25	+4.3387E-005	+0.0014	+0.9891	26	+4.1767E-005	+0.0014	+0.9905
27	+3.7682E-005	+0.0012	+0.9917	28	+3.3319E-005	+0.0011	+0.9928
29	+3.0305E-005	+0.0010	+0.9938	30	+2.8190E-005	+0.0009	+0.9947
31	+2.5782E-005	+0.0008	+0.9956	32	+2.3218E-005	+0.0008	+0.9964
33	+2.1109E-005	+0.0007	+0.9970	34	+1.7890E-005	+0.0006	+0.9976
35	+1.6137E-005	+0.0005	+0.9982	36	+1.3459E-005	+0.0004	+0.9986
37	+1.2224E-005	+0.0004	+0.9990	38	+1.0128E-005	+0.0003	+0.9993
39	+8.0348E-006	+0.0003	+0.9996	40	+6.5610E-006	+0.0002	+0.9998

Table S2. The relative synonymous codon usage (RSCU) patterns of ICV *M* gene and its hosts.

AA	Codon	M gene	Swine	Bovine	Human
**Phe**	**UUU(F)**	**1.58**	0.79	0.85	0.93
	**UUC(F)**	0.42	**1.21**	**1.15**	**1.07**
**Leu**	**UUA(L)**	**1.52**	0.32	0.38	0.46
	**UUG(L)**	1.41	0.67	0.71	0.77
	**CUU(L)**	0.75	1.35	0.70	0.79
	**CUC(L)**	0.52	1.35	1.26	1.17
	**CUA(L)**	1.25	0.33	0.36	0.43
	**CUG(L)**	0.55	**2.68**	**2.59**	**2.37**
**Ile**	**AUU(I)**	1.22	0.91	0.98	1.08
	**AUC(I)**	0.45	**1.67**	**1.57**	**1.41**
	**AUA(I)**	**1.34**	0.42	0.45	0.51
**Val**	**GUU(V)**	**1.99**	0.57	0.64	0.73
	**GUC(V)**	0.52	1.07	1.01	0.95
	**GUA(V)**	0.98	0.34	0.40	0.47
	**GUG(V)**	0.52	**2.03**	**1.95**	**1.85**
**Ser**	**UCU(S)**	1.67	0.99	1.04	1.13
	**UCC(S)**	0.25	1.50	1.37	1.31
	**UCA(S)**	1.66	0.73	0.79	0.90
	**UCG(S)**	0.27	0.39	0.39	0.33
	**AGU(S)**	**1.68**	0.77	0.87	0.90
	**AGC(S)**	0.48	**1.62**	**1.53**	**1.44**
**Pro**	**CCU(P)**	**1.94**	1.05	1.08	1.15
	**CCC(P)**	0.71	**1.46**	**1.39**	**1.29**
	**CCA(P)**	1.34	0.94	1.00	1.11
	**CCG(P)**	0.01	0.56	0.53	0.45

**Thr**	**ACU(T)**	0.98	0.83	0.89	0.99
	**ACC(T)**	0.87	**1.68**	**1.55**	**1.42**
	**ACA(T)**	**2.14**	0.92	1.01	1.14
	**ACG(T)**	0.02	0.57	0.56	0.46
**Ala**	**GCU(A)**	1.21	0.96	1.00	1.06
	**GCC(A)**	1	**1.80**	**1.71**	**1.60**
	**GCA(A)**	**1.69**	0.74	0.80	0.91
	**GCG(A)**	0.11	0.50	0.48	0.42
**Tyr**	**UAU(Y)**	**1.11**	0.73	0.79	0.89
	**UAC(Y)**	0.89	**1.27**	**1.21**	**1.11**
**His**	**CAU(H)**	**1.02**	0.70	0.75	0.84
	**CAC(H)**	0.98	**1.30**	**1.25**	**1.16**
**Gln**	**CAA(Q)**	**1.98**	0.44	0.46	0.53
	**CAG(Q)**	0.02	**1.56**	**1.54**	**1.47**
**Asn**	**AAU(N)**	**1.32**	0.79	0.81	1.08
	**AAC(N)**	0.68	**1.21**	**1.19**	**1.41**
**Lys**	**AAA(K)**	**1.53**	0.76	0.78	0.87
	**AAG(K)**	0.47	**1.24**	**1.22**	**1.13**
**Asp**	**GAU(D)**	0.79	0.80	0.84	0.93
	**GAC(D)**	**1.21**	**1.20**	**1.16**	**1.07**
**Glu**	**GAA(E)**	**1.5**	0.72	0.78	0.84
	**GAG(E)**	0.5	**1.28**	**1.22**	**1.16**
**Cys**	**UGU(C)**	0.79	0.79	0.85	0.91
	**UGC(C)**	**1.21**	**1.21**	**1.15**	**1.09**
**Arg**	**CGU(R)**	0	0.44	0.49	0.48
	**CGC(R)**	0	**1.31**	1.17	1.10
	**CGA(R)**	0.6	0.60	0.68	0.65
	**CGG(R)**	0	1.29	**1.32**	1.21
	**AGA(R)**	**3.9**	1.12	1.14	**1.29**
	**AGG(R)**	1.5	1.23	1.20	1.27
**Gly**	**GGU(G)**	0.65	0.57	0.64	0.65
	**GGC(G)**	0.51	**1.46**	**1.43**	**1.35**
	**GGA(G)**	**2.6**	0.91	0.95	1.00
	**GGG(G)**	0.25	1.05	0.99	1.00

Preferred codons of overall ICV *M* gene and potential hosts are shown in bold.

Table S3. The site-by-site selection analysis results of the rest of amino acids

**Gene**	**AA**	**FEL**		**SLAC**		**FUBAR**		**MEME**	
	**Site**	**dN-dS**	**p-value**	**dN-dS**	**p-value**	**dN-dS**	**Post.Pro**	**dN^{+}- dS**	**p-value**
HE	**1**	-1.185	0.066	-1.912	1.0000	-2.238	0.028	-1.18	0.67
	**2**	0.26	0.522	0.718	0.7270	-0.362	0.411	0.37	0.45
	**3**	0.231	0.701	0.547	0.9329	-0.792	0.321	47.08	0.37
	**4**	-0.858	0.049	-3.483	1.0000	-2.192	0.018	-0.86	0.67
	**5**	0	1	0.000	1.0000	-1.562	0.1	0	1
	**6**	0	1	0.000	1.0000	-0.47	0.251	0	1
	**7**	0	1	0.000	1.0000	-0.774	0.184	0	1
	**8**	0	1	0.000	1.0000	-1.013	0.19	0	1
	**9**	0	1	0.000	1.0000	-0.5	0.246	0	1
	**10**	0	1	0.000	1.0000	-1.013	0.19	0	1
	**11**	-0.379	0.194	-1.531	1.0000	-1.35	0.072	-0.38	0.67
	**12**	-1.283	0.023	-4.077	1.0000	-2.195	0.017	-1.28	0.67
	**13**	0	1	0.000	1.0000	-0.863	0.175	0	1

**14**	0	1	0.000	1.0000	-0.517	0.224	0	1
**15**	-0.614	0.147	-2.045	1.0000	-1.377	0.071	-0.61	0.67
**16**	0	1	0.000	1.0000	-0.517	0.224	0	1
**17**	-0.366	0.301	-0.876	1.0000	-0.848	0.137	-0.37	0.67
**18**	-1.24	0.024	-4.090	1.0000	-2.193	0.015	-1.24	0.67
**19**	0.258	0.503	0.691	0.7385	-0.171	0.463	2018.18	0.11
**20**	-0.236	0.62	-0.765	0.8889	-0.467	0.279	-0.47	0.67
**21**	-5.293	0	-11.966	1.0000	-5.223	0	-5.29	0.67
**22**	-0.643	0.372	-0.735	0.8830	-1.297	0.157	-0.89	0.67
**23**	0	1	0.000	1.0000	-0.969	0.167	0	1
**24**	-1.048	0.072	-1.952	1.0000	-1.889	0.041	-1.05	0.67
**25**	-0.354	0.313	-0.929	1.0000	-0.944	0.129	-0.35	0.67
**26**	-1.025	0.105	-1.954	1.0000	-1.823	0.051	-1.03	0.67
**27**	-0.46	0.15	-1.531	1.0000	-1.2	0.076	-0.46	0.67
**28**	-2.678	0.003	-4.592	1.0000	-3.347	0.001	-2.68	0.67
**29**	0	1	0.000	1.0000	-1.117	0.155	0	1
**30**	0	1	0.000	1.0000	-1.117	0.155	0	1
**31**	0.261	0.522	0.718	0.7269	-0.362	0.412	0.46	0.45
**32**	0	1	0.000	1.0000	-1.07	0.152	0	1
**33**	0	1	0.000	1.0000	-2.434	0.064	0	1
**34**	0	1	0.000	1.0000	-0.812	0.196	0	1
**35**	-1.991	0.004	-7.046	1.0000	-3.029	0.001	-1.99	0.67
**36**	-0.823	0.101	-1.531	1.0000	-1.813	0.039	-0.82	0.67
**37**	-2.826	0.002	-6.123	1.0000	-3.084	0.001	-2.83	0.67
**38**	-0.43	0.164	-1.741	1.0000	-1.432	0.069	-0.43	0.67
**39**	-2.228	0.013	-6.889	0.9986	-3.05	0.002	-2.5	0.67
**40**	-1.122	0.068	-1.907	1.0000	-2.208	0.029	-1.12	0.67
**41**	0.508	0.297	1.531	0.4444	0.114	0.606	49.9	0.17
**42**	0	1	0.000	1.0000	-0.969	0.167	0	1
**43**	-1.509	0.01	-4.592	1.0000	-2.53	0.004	-1.51	0.67
**44**	-0.709	0.132	-1.531	1.0000	-1.643	0.053	-0.71	0.67
**45**	-2.928	0.001	-9.185	1.0000	-3.312	0	-2.93	0.67
**46**	-1.328	0.049	-3.687	1.0000	-2.575	0.004	-1.33	0.67
**47**	-1.051	0.072	-1.952	1.0000	-1.891	0.041	-1.05	0.67
**48**	-2.567	0.019	-3.809	1.0000	-3.09	0.008	-2.57	0.67
**49**	-3.213	0.001	-5.865	1.0000	-3.02	0.001	-3.21	0.67
**50**	0.254	0.465	0.765	0.6667	-0.296	0.417	0.38	0.42
**51**	0	1	0.000	1.0000	-2.4	0.133	0	1
**52**	-0.882	0.092	-1.720	1.0000	-1.802	0.044	-0.88	0.67
**53**	-0.234	0.621	-0.768	0.8892	-0.465	0.28	-0.47	0.67
**54**	0	1	0.000	1.0000	-0.812	0.196	0	1
**55**	-0.829	0.083	-1.531	1.0000	-2.039	0.033	-0.83	0.67
**56**	-1.053	0.071	-1.952	1.0000	-1.89	0.041	-1.05	0.67
**57**	-0.84	0.134	-1.531	1.0000	-2.02	0.037	-0.84	0.67
**58**	-2.302	0.001	-7.675	1.0000	-3.352	0	-2.3	0.67
**59**	0	1	0.000	1.0000	-0.767	0.187	0	1
**60**	-0.618	0.11	-2.042	1.0000	-1.393	0.068	-0.62	0.67
**61**	0.549	0.354	1.495	0.5164	0.004	0.565	35.62	0.21
**62**	0.654	0.164	2.087	0.3949	0.54	0.767	2.27	0.18
**63**	-1.051	0.072	-1.952	1.0000	-1.891	0.041	-1.05	0.67
**64**	-1.385	0.186	-2.211	0.9544	-2.454	0.046	-1.41	0.67
**65**	1.041	0.144	2.997	0.2151	0.998	0.811	**1670.22**	**0**
**66**	0	1	0.000	1.0000	-0.812	0.196	0	1
**67**	-3.068	0.001	-6.123	1.0000	-3.188	0	-3.07	0.67
**68**	-1.737	0.045	-6.060	0.9928	-3.588	0.004	-2.23	0.67
**69**	-1.697	0.025	-3.062	1.0000	-2.472	0.01	-1.7	0.67

**70**	-2.819	0.001	-7.654	1.0000	-3.713	0	-2.82	0.67
**71**	0	1	0.000	1.0000	-0.822	0.192	0	1
**72**	-0.821	0.101	-1.531	1.0000	-1.772	0.046	-0.82	0.67
**73**	-0.857	0.13	-1.531	1.0000	-2.008	0.037	-0.86	0.67
**74**	-0.808	0.538	-0.205	0.7086	-1.489	0.186	-1.43	0.67
**75**	-2.505	0.026	-4.905	0.9940	-3.954	0.01	-2.51	0.67
**76**	-0.652	0.384	-0.765	0.8889	-1.035	0.178	-0.69	0.67
**77**	-1.43	0.021	-3.062	1.0000	-2.375	0.011	-1.43	0.67
**78**	1.042	0.178	2.766	0.2968	1.225	0.821	1.04	0.2
**79**	-0.982	0.04	-3.544	1.0000	-2.304	0.014	-0.98	0.67
**80**	-1.005	0.103	-1.960	1.0000	-1.841	0.045	-1.01	0.67
**81**	-1.265	0.114	-2.300	0.9631	-1.791	0.064	-1.5	0.67
**82**	-0.487	0.535	-0.766	0.8890	-0.921	0.203	-0.84	0.67
**83**	-0.924	0.332	-2.484	0.9388	-2.1	0.027	-1.45	0.67
**84**	-0.468	0.544	-0.765	0.8889	-0.895	0.207	-0.58	0.67
**85**	-0.818	0.081	-1.531	1.0000	-1.784	0.044	-0.82	0.67
**86**	-2.403	0.003	-4.592	1.0000	-3.194	0.001	-2.4	0.67
**87**	-1.54	0.018	-4.592	1.0000	-2.531	0.005	-1.54	0.67
**88**	-1.589	0.036	-3.739	1.0000	-2.678	0.003	-1.59	0.67
**89**	0	1	0.000	1.0000	-2.434	0.064	0	1
**90**	-1.538	0.033	-4.554	1.0000	-2.69	0.001	-1.54	0.67
**91**	0	1	0.000	1.0000	-0.863	0.175	0	1
**92**	0	1	0.000	1.0000	-2.434	0.064	0	1
**93**	0	1	0.000	1.0000	-0.812	0.196	0	1
**94**	-2.411	0.001	-7.654	1.0000	-2.945	0	-2.41	0.67
**95**	-2.868	0.002	-4.592	1.0000	-3.343	0.001	-2.87	0.67
**96**	-0.825	0.089	-1.531	1.0000	-1.841	0.035	-0.82	0.67
**97**	-0.379	0.161	-1.531	1.0000	-1.367	0.068	-0.38	0.67
**98**	-0.986	0.04	-3.547	1.0000	-2.304	0.014	-0.99	0.67
**99**	-1.151	0.29	-1.536	0.8893	-2.196	0.092	-1.16	0.67
**100**	-3.819	0	-10.074	1.0000	-3.548	0	-3.82	0.67
**101**	-2.042	0.013	-6.077	1.0000	-2.62	0.005	-2.04	0.67
**102**	-0.826	0.101	-1.531	1.0000	-1.818	0.039	-0.83	0.67
**103**	-0.665	0.153	-2.026	1.0000	-1.41	0.078	-0.66	0.67
**104**	0	1	0.000	1.0000	-1.013	0.19	0	1
**105**	0	1	0.000	1.0000	-0.969	0.167	0	1
**106**	0	1	0.000	1.0000	-0.819	0.193	0	1
**107**	-0.845	0.081	-1.531	1.0000	-2.059	0.032	-0.84	0.67
**108**	-0.992	0.077	-1.890	1.0000	-2.13	0.031	-0.99	0.67
**109**	-1.05	0.072	-1.952	1.0000	-1.926	0.035	-1.05	0.67
**110**	-3.347	0	-9.053	1.0000	-4.57	0	-3.34	0.67
**111**	-1.688	0.02	-3.062	1.0000	-2.47	0.01	-1.69	0.67
**112**	0	1	0.000	1.0000	-0.812	0.196	0	1
**113**	0	1	0.000	1.0000	-0.772	0.212	0	1
**114**	-1.188	0.066	-1.912	1.0000	-2.24	0.028	-1.19	0.67
**115**	0	1	0.000	1.0000	-0.969	0.167	0	1
**116**	-1.935	0.008	-4.592	1.0000	-2.678	0.003	-1.94	0.67
**117**	-0.371	0.519	-1.365	0.9377	-0.591	0.256	-0.63	0.67
**118**	0	1	0.000	1.0000	-2.4	0.133	0	1
**119**	-6.213	0	-11.818	1.0000	-6.349	0	-6.21	0.67
**120**	0.254	0.465	0.765	0.6667	-0.335	0.396	0.33	0.42
**121**	-0.494	0.609	-0.525	0.8240	-0.755	0.29	-0.6	0.67
**122**	0.253	0.461	0.765	0.6667	-0.158	0.459	0.32	0.42
**123**	0	1	0.000	1.0000	-0.764	0.188	0	1
**124**	-4.61	0.002	-9.753	1.0000	-3.794	0.003	-4.61	0.67
**125**	1.338	0.13	3.455	0.2197	2.107	0.884	1.34	0.16

**126**	-1.019	0.081	-1.956	1.0000	-1.861	0.043	-1.02	0.67
**127**	-0.612	0.164	-2.045	1.0000	-1.318	0.085	-0.61	0.67
**128**	0.449	0.377	1.500	0.4805	-0.03	0.555	0.49	0.36
**129**	-1.787	0.015	-3.753	1.0000	-2.737	0.007	-1.79	0.67
**130**	-3.197	0.002	-5.871	1.0000	-3.017	0.002	-3.2	0.67
**131**	0	1	0.000	1.0000	-2.4	0.133	0	1
**132**	-1.433	0.021	-3.062	1.0000	-2.38	0.01	-1.43	0.67
**133**	-4.094	0	-10.004	1.0000	-3.732	0	-4.09	0.67
**134**	-1.658	0.011	-4.921	1.0000	-2.569	0.004	-1.66	0.67
**135**	-0.818	0.092	-1.864	1.0000	-2.005	0.036	-0.82	0.67
**136**	-1.377	0.039	-4.426	1.0000	-2.617	0.002	-1.38	0.67
**137**	0	1	0.000	1.0000	-0.825	0.19	0	1
**138**	-1.239	0.04	-4.090	1.0000	-2.156	0.019	-1.24	0.67
**139**	0	1	0.000	1.0000	-0.411	0.294	0	1
**140**	0.26	0.522	0.718	0.7269	-0.362	0.411	0.34	0.45
**141**	0.713	0.16	2.040	0.4228	0.624	0.777	0.71	0.18
**142**	-0.759	0.106	-1.701	1.0000	-1.679	0.05	-0.76	0.67
**143**	0	1	0.000	1.0000	-0.772	0.212	0	1
**144**	0	1	0.000	1.0000	-1.117	0.155	0	1
**145**	-0.65	0.167	-1.840	1.0000	-1.64	0.052	-0.65	0.67
**146**	-1.665	0.016	-3.062	1.0000	-2.476	0.009	-1.67	0.67
**147**	0	1	0.000	1.0000	-0.934	0.18	0	1
**148**	0.372	0.453	0.967	0.6643	-0.248	0.455	0.37	0.41
**149**	-0.837	0.135	-1.531	1.0000	-2.017	0.037	-0.84	0.67
**150**	0	1	0.000	1.0000	-0.865	0.174	0	1
**151**	0	1	0.000	1.0000	-0.47	0.251	0	1
**152**	-2.274	0.035	-7.795	0.9982	-2.841	0.028	-2.51	0.67
**153**	-0.612	0.096	-2.045	1.0000	-1.424	0.059	-0.61	0.67
**154**	-1.945	0.02	-4.660	1.0000	-2.875	0.001	-1.94	0.67
**155**	-0.883	0.054	-3.062	1.0000	-2.259	0.015	-0.88	0.67
**156**	0	1	0.000	1.0000	-0.825	0.19	0	1
**157**	-2.823	0.006	-6.889	0.9986	-3.871	0.001	-3.12	0.67
**158**	-0.32	0.331	-0.920	1.0000	-0.835	0.141	-0.32	0.67
**159**	-0.629	0.108	-2.042	1.0000	-1.414	0.067	-0.63	0.67
**160**	-2.459	0.009	-3.821	1.0000	-3.031	0.005	-2.46	0.67
**161**	-0.82	0.09	-1.531	1.0000	-1.804	0.04	-0.82	0.67
**162**	-1.303	0.146	-2.669	0.9729	-1.769	0.073	-1.62	0.67
**163**	-2.766	0.001	-6.123	1.0000	-3.182	0	-2.77	0.67
**164**	-0.619	0.223	-0.966	1.0000	-1.499	0.079	-0.62	0.67
**165**	0.7	0.666	2.956	0.3380	2.117	0.652	1.19	0.53
**166**	-0.016	0.986	-1.531	0.8267	-0.679	0.201	-0.02	0.67
**167**	0	1	0.000	1.0000	-0.767	0.187	0	1
**168**	-0.68	0.641	0.407	0.6246	-0.677	0.268	-4.24	0.67
**169**	-1.708	0.004	-6.123	1.0000	-2.906	0.001	-1.71	0.67
**170**	0.591	0.265	1.531	0.4445	0.185	0.628	**90.21**	**0**
**171**	0.253	0.466	0.766	0.6666	-0.292	0.418	0.36	0.42
**172**	1.233	0.426	4.205	0.2186	4.348	0.832	**13.73**	**0.01**
**173**	0	1	0.000	1.0000	-0.825	0.19	0	1
**174**	0	1	0.000	1.0000	-0.47	0.251	0	1
**175**	0	1	0.000	1.0000	-0.487	0.241	0	1
**177**	-1.354	0.016	-4.053	1.0000	-2.266	0.013	-1.35	0.67
**178**	-0.613	0.147	-2.045	1.0000	-1.376	0.071	-0.61	0.67
**179**	-2.29	0.018	-3.785	1.0000	-2.922	0.001	-2.29	0.67
**180**	-1.183	0.065	-1.912	1.0000	-2.24	0.028	-1.18	0.67
**181**	0	1	0.000	1.0000	-0.825	0.19	0	1
**182**	-0.86	0.086	-1.531	1.0000	-2.068	0.026	-0.86	0.67

**183**	-1.717	0.015	-3.062	1.0000	-2.723	0.006	-1.72	0.67
**184**	-1.693	0.027	-4.572	1.0000	-2.742	0.001	-1.69	0.67
**185**	-1.253	0.048	-4.089	1.0000	-2.126	0.023	-1.25	0.67
**186**	0	1	0.000	1.0000	-0.996	0.157	0	1
**187**	0	1	0.000	1.0000	-1.013	0.19	0	1
**188**	-1.342	0.376	-1.988	0.8415	-2.153	0.109	-1.17	0.67
**189**	-2.56	0.001	-7.654	1.0000	-3.017	0	-2.56	0.67
**190**	-0.269	0.869	0.717	0.5893	0.756	0.427	-0.36	0.67
**191**	0	1	0.000	1.0000	-0.802	0.172	0	1
**192**	0.356	0.742	1.267	0.4965	-0.242	0.424	0.36	0.57
**193**	0.719	0.587	2.495	0.3733	1.944	0.655	0.72	0.49
**195**	0.257	0.539	0.692	0.7527	-0.402	0.401	0.27	0.47
**196**	-1.027	0.081	-1.955	1.0000	-1.867	0.043	-1.03	0.67
**197**	-2.262	0.001	-7.654	1.0000	-3.386	0	-2.26	0.67
**199**	-1.811	0.015	-3.754	1.0000	-2.74	0.007	-1.81	0.67
**200**	0	1	0.000	1.0000	-0.825	0.19	0	1
**201**	-0.823	0.101	-1.531	1.0000	-1.815	0.039	-0.82	0.67
**202**	0.253	0.461	0.765	0.6667	-0.158	0.459	0.32	0.42
**203**	-2.069	0.095	-6.168	0.9934	-2.767	0.018	-2.74	0.67
**204**	0	1	0.000	1.0000	-0.476	0.247	0	1
**205**	0.77	0.206	2.290	0.2988	0.312	0.671	3.82	0.21
**206**	-1.666	0.02	-3.062	1.0000	-2.481	0.008	-1.67	0.67
**207**	-1.749	0.015	-3.062	1.0000	-2.744	0.005	-1.75	0.67
**208**	-1.357	0.081	-3.828	0.9877	-2.067	0.032	-1.62	0.67
**209**	0.427	0.402	1.105	0.6155	-0.152	0.481	1	0.38
**210**	0	1	0.000	1.0000	-0.812	0.196	0	1
**211**	-0.839	0.082	-1.531	1.0000	-2.047	0.033	-0.84	0.67
**212**	-0.46	0.15	-1.531	1.0000	-1.204	0.075	-0.46	0.67
**213**	-1.051	0.102	-1.952	1.0000	-1.843	0.05	-1.05	0.67
**214**	-0.809	0.314	-3.759	0.9696	-1.825	0.071	-1.36	0.67
**215**	0	1	0.000	1.0000	-0.47	0.251	0	1
**216**	0.779	0.262	2.180	0.3709	0.403	0.673	0.78	0.27
**217**	0.424	0.573	0.495	0.5969	0.201	0.491	0.43	0.48
**218**	0	1	0.000	1.0000	-0.865	0.174	0	1
**219**	0	1	0.000	1.0000	-0.774	0.184	0	1
**220**	0	1	0.000	1.0000	-0.466	0.247	0	1
**221**	0	1	0.000	1.0000	-0.764	0.188	0	1
**222**	0	1	0.000	1.0000	-0.735	0.201	0	1
**223**	-0.613	0.13	-2.045	1.0000	-1.368	0.073	-0.61	0.67
**224**	0	1	0.000	1.0000	-0.772	0.212	0	1
**225**	-0.617	0.146	-2.044	1.0000	-1.383	0.071	-0.62	0.67
**226**	-1.857	0.009	-4.982	1.0000	-2.638	0.003	-1.86	0.67
**227**	0	1	0.000	1.0000	-0.772	0.212	0	1
**228**	-1.019	0.073	-1.956	1.0000	-1.865	0.043	-1.02	0.67
**229**	-2.988	0.001	-5.902	1.0000	-2.942	0.002	-2.99	0.67
**230**	-2.479	0.009	-3.824	1.0000	-3.041	0.005	-2.48	0.67
**231**	0	1	0.000	1.0000	-1.117	0.155	0	1
**232**	0	1	0.000	1.0000	-0.466	0.247	0	1
**233**	-2.093	0.012	-6.084	1.0000	-2.639	0.005	-2.09	0.67
**234**	-1.049	0.072	-1.952	1.0000	-1.89	0.041	-1.05	0.67
**235**	0	1	0.000	1.0000	-1.12	0.153	0	1
**236**	-2.488	0.025	-3.723	0.9839	-3.959	0.009	-2.6	0.67
**237**	-0.885	0.078	-1.531	1.0000	-2.085	0.031	-0.88	0.67
**238**	-1.025	0.081	-1.954	1.0000	-1.866	0.043	-1.03	0.67
**239**	0.993	0.08	2.859	0.2599	1.334	0.882	0.99	0.1
**240**	0.483	0.247	1.360	0.5633	0.273	0.672	0.48	0.26

**241**	-0.612	0.164	-2.045	1.0000	-1.319	0.085	-0.61	0.67
**242**	0	1	0.000	1.0000	-0.812	0.196	0	1
**243**	0	1	0.000	1.0000	-1.013	0.19	0	1
**244**	0	1	0.000	1.0000	-0.466	0.247	0	0.67
**245**	-0.971	0.046	-3.245	1.0000	-2.014	0.023	-0.97	0.67
**246**	-1.023	0.105	-1.955	1.0000	-1.821	0.051	-1.02	0.67
**247**	-1.066	0.07	-1.952	1.0000	-1.899	0.041	-1.07	0.67
**248**	-0.028	0.979	0.216	0.7000	-0.795	0.296	-0.04	0.67
**249**	1.719	0.062	3.826	0.1322	1.967	0.892	**84.59**	**0.03**
**250**	0	1	0.000	1.0000	-0.969	0.167	0	1
**251**	-2.485	0.009	-3.824	1.0000	-3.044	0.005	-2.48	0.67
**252**	-0.507	0.689	-0.020	0.6832	-0.475	0.296	-0.76	0.67
**253**	-0.821	0.101	-1.531	1.0000	-1.772	0.046	-0.82	0.67
**254**	-0.837	0.083	-1.531	1.0000	-2.045	0.033	-0.84	0.67
**255**	-3.741	0	-9.185	1.0000	-5.909	0	-3.74	0.67
**256**	-0.366	0.301	-0.876	1.0000	-0.847	0.138	-0.37	0.67
**257**	0	1	0.000	1.0000	-0.822	0.192	0	1
**258**	0	1	0.000	1.0000	-0.487	0.241	0	1
**259**	-0.187	0.669	-0.747	0.8867	-0.622	0.246	-0.41	0.67
**260**	-0.36	0.703	0.000	0.7407	-0.965	0.265	-0.43	0.67
**261**	0	1	0.000	1.0000	-0.934	0.18	0	1
**262**	0.254	0.46	0.765	0.6667	-0.198	0.436	0.41	0.41
**263**	0	1	0.000	1.0000	-0.432	0.273	0	1
**264**	0	1	0.000	1.0000	-0.996	0.157	0	1
**265**	-2.653	0	-9.185	1.0000	-4.034	0	-2.65	0.67
**266**	-2.158	0.011	-3.904	1.0000	-2.569	0.009	-2.16	0.67
**267**	-0.143	0.879	0.000	0.7407	-0.54	0.336	-0.36	0.67
**268**	-0.803	0.086	-1.531	1.0000	-2.019	0.034	-0.8	0.67
**269**	-0.374	0.514	-1.365	0.9377	-0.588	0.258	-0.62	0.67
**270**	-0.692	0.402	-1.533	0.8890	-1.145	0.169	-1.22	0.67
**271**	0	1	0.000	1.0000	-1.033	0.144	0	1
**272**	-1.663	0.016	-3.062	1.0000	-2.702	0.006	-1.66	0.67
**273**	-1338	0.007	-12.403	0.9721	-10.406	0.163	-1257.21	0.67
**274**	0	1	0.000	1.0000	-1.013	0.19	0	1
**275**	0	1	0.000	1.0000	-0.819	0.193	0	1
**276**	-0.611	0.111	-2.045	1.0000	-1.383	0.069	-0.61	0.67
**277**	0	1	0.000	1.0000	-2.434	0.064	0	1
**278**	0.565	0.566	0.636	0.5904	0.807	0.565	0.57	0.48
**279**	0	1	0.000	1.0000	-0.411	0.294	0	1
**280**	-1.949	0.015	-6.244	1.0000	-2.853	0	-1.95	0.67
**281**	-2.696	0.007	-3.816	1.0000	-3.128	0.005	-2.69	0.67
**282**	-0.83	0.089	-1.531	1.0000	-1.846	0.034	-0.83	0.67
**283**	-0.777	0.088	-1.531	1.0000	-1.997	0.034	-0.78	0.67
**284**	-0.99	0.067	-1.642	1.0000	-2.163	0.027	-0.99	0.67
**285**	-1.049	0.103	-1.952	1.0000	-1.842	0.05	-1.05	0.67
**286**	0	1	0.000	1.0000	-0.934	0.18	0	1
**287**	0	1	0.000	1.0000	-0.96	0.171	0	1
**288**	0	1	0.000	1.0000	-1.07	0.152	0	1
**289**	-1.238	0.018	-4.090	1.0000	-2.191	0.015	-1.24	0.67
**290**	0.336	0.405	0.765	0.6667	-0.124	0.476	345.24	0.18
**291**	-0.677	0.107	-1.531	1.0000	-1.957	0.03	-0.68	0.67
**292**	-0.98	0.068	-1.640	1.0000	-2.156	0.027	-0.98	0.67
**293**	-2.032	0.01	-6.077	1.0000	-2.634	0.004	-2.03	0.67
**294**	-0.817	0.102	-1.531	1.0000	-1.808	0.039	-0.82	0.67
**295**	-0.603	0.227	-0.965	1.0000	-1.477	0.081	-0.6	0.67
**296**	0.164	0.969	0.511	0.9993	-2.287	0.237	-0.19	0.67

**297**	-1.855	0.013	-3.062	1.0000	-2.582	0.006	-1.86	0.67
**298**	-1.763	0.016	-3.747	1.0000	-2.719	0.007	-1.76	0.67
**299**	-2.544	0.001	-6.333	1.0000	-3.422	0	-2.54	0.67
**300**	-1.844	0.01	-3.942	1.0000	-2.482	0.009	-1.84	0.67
**301**	-2.188	0.02	-3.910	1.0000	-2.555	0.011	-2.19	0.67
**302**	-0.619	0.129	-2.043	1.0000	-1.379	0.072	-0.62	0.67
**303**	0	1	0.000	1.0000	-0.812	0.196	0	1
**304**	-0.614	0.111	-2.045	1.0000	-1.388	0.069	-0.61	0.67
**305**	0	1	0.000	1.0000	-2.434	0.064	0	1
**306**	0	1	0.000	1.0000	-1.12	0.153	0	1
**307**	0	1	0.000	1.0000	-1.117	0.155	0	1
**308**	-0.887	0.318	-1.179	0.9244	-1.53	0.142	-0.93	0.67
**309**	0	1	0.000	1.0000	-0.969	0.167	0	1
**310**	-0.587	0.414	-0.756	0.8878	-1.325	0.137	-0.85	0.67
**311**	-0.937	0.049	-3.062	1.0000	-1.989	0.024	-0.94	0.67
**312**	0	1	0.000	1.0000	-0.767	0.187	0	1
**313**	-1.674	0.016	-3.062	1.0000	-2.478	0.009	-1.67	0.67
**314**	-1.142	0.131	-3.852	0.9879	-1.87	0.045	-1.45	0.67
**315**	-1.186	0.101	-1.912	1.0000	-2.172	0.04	-1.19	0.67
**316**	-1.918	0.008	-6.123	1.0000	-2.727	0.001	-1.92	0.67
**317**	0.704	0.693	1.596	0.4763	3.088	0.639	0.71	0.55
**318**	0	1	0.000	1.0000	-2.4	0.133	0	1
**319**	-1.509	0.017	-3.062	1.0000	-2.56	0.008	-1.51	0.67
**320**	0	1	0.000	1.0000	-1.12	0.153	0	1
**321**	0.209	0.749	0.627	0.6227	0.212	0.569	0.21	0.57
**322**	-2.024	0.009	-3.281	1.0000	-2.855	0.005	-2.02	0.67
**323**	1.561	0.123	3.671	0.1971	2.51	0.858	1.56	0.15
**324**	-0.989	0.382	-1.532	0.8890	-1.513	0.136	-2.61	0.67
**325**	-0.04	0.962	-0.456	0.7738	-0.07	0.474	-1.88	0.67
**326**	1.568	0.11	3.049	0.2671	2.056	0.867	**1022.88**	**0**
**327**	0	1	0.000	1.0000	-0.993	0.158	0	1
**328**	-2.176	0.003	-7.654	1.0000	-3.306	0	-2.18	0.67
**329**	0	1	0.000	1.0000	-0.822	0.192	0	1
**330**	-4.172	0.001	-9.280	1.0000	-6.096	0	-4.17	0.67
**331**	-1.681	0.016	-3.062	1.0000	-2.482	0.009	-1.68	0.67
**332**	0.872	0.126	2.234	0.3940	0.763	0.797	12.95	0.11
**333**	-1	0.309	-2.480	0.9382	-2.171	0.024	-1.55	0.67
**334**	0	1	0.000	1.0000	-0.517	0.224	0	1
**335**	0	1	0.000	1.0000	-0.767	0.187	0	1
**336**	0	1	0.000	1.0000	-1.033	0.144	0	1
**337**	-1.546	0.005	-6.123	1.0000	-2.819	0.001	-1.54	0.67
**338**	-1.903	0.008	-6.123	1.0000	-2.589	0.004	-1.9	0.67
**339**	0	1	0.000	1.0000	-2.434	0.064	0	1
**340**	-2.37	0.011	-5.566	1.0000	-3.181	0	-2.37	0.67
**341**	-1.931	0.064	-6.644	0.9951	-2.843	0.008	-2.54	0.67
**342**	0	1	0.000	1.0000	-1.013	0.19	0	1
**343**	-0.486	0.147	-1.772	1.0000	-1.563	0.06	-0.49	0.67
**344**	0.412	0.433	1.051	0.6453	-0.209	0.467	0.98	0.4
**345**	-0.956	0.253	-3.757	0.9699	-2.051	0.058	-1.49	0.67
**346**	-1.368	0.023	-3.062	1.0000	-2.585	0.006	-1.37	0.67
**347**	-1.533	0.127	-5.360	0.9945	-2.038	0.042	-1.61	0.67
**348**	0.852	0.499	2.813	0.3049	1.784	0.713	**2033.2**	**0.02**
**349**	-1.756	0.013	-3.062	1.0000	-2.735	0.006	-1.76	0.67
**350**	-1.987	0.013	-3.776	1.0000	-2.819	0.006	-1.99	0.67
**351**	0.508	0.297	1.531	0.4444	0.114	0.606	19.38	0.22
**352**	-2.87	0.002	-5.901	1.0000	-2.908	0.002	-2.87	0.67

**353**	-0.783	0.343	-1.247	0.9308	-1.038	0.19	-1.05	0.67
**354**	-3.068	0.001	-8.040	1.0000	-3.045	0	-3.07	0.67
**355**	-1.052	0.072	-1.952	1.0000	-1.927	0.035	-1.05	0.67
**356**	-0.816	0.084	-1.531	1.0000	-2.029	0.033	-0.82	0.67
**357**	0	1	0.000	1.0000	-0.822	0.192	0	1
**358**	1.221	0.179	3.087	0.2740	1.273	0.756	5.5	0.19
**359**	0	1	0.000	1.0000	-1.117	0.155	0	1
**360**	0	1	0.000	1.0000	-2.434	0.064	0	1
**361**	-1.236	0.012	-4.555	1.0000	-2.596	0.004	-1.24	0.67
**362**	-0.615	0.312	-2.749	0.9731	-1.421	0.105	-0.89	0.67
**363**	-0.97	0.091	-2.758	1.0000	-2.246	0.016	-0.97	0.67
**364**	-0.256	0.365	-0.887	1.0000	-0.572	0.17	-0.26	0.67
**365**	-0.848	0.133	-1.531	1.0000	-2.026	0.037	-0.85	0.67
**366**	-4.464	0.003	-6.440	0.9874	-10.186	0	-5.21	0.67
**367**	-0.723	0.13	-2.627	1.0000	-1.787	0.034	-0.72	0.67
**368**	-1.002	0.164	-3.409	0.9845	-1.363	0.107	-1.25	0.67
**369**	-1.046	0.103	-1.952	1.0000	-1.839	0.05	-1.05	0.67
**370**	0	1	0.000	1.0000	-1.117	0.155	0	1
**371**	-0.817	0.09	-1.531	1.0000	-1.798	0.041	-0.82	0.67
**372**	0	1	0.000	1.0000	-1.07	0.152	0	1
**373**	-2.047	0.007	-6.079	1.0000	-2.64	0.004	-2.05	0.67
**374**	0.489	0.485	1.171	0.7602	-0.291	0.49	0.55	0.43
**375**	-2.273	0.008	-6.123	1.0000	-3.282	0.001	-2.27	0.67
**376**	0	1	0.000	1.0000	-1.013	0.19	0	1
**377**	0	1	0.000	1.0000	-0.934	0.18	0	1
**378**	-0.883	0.038	-3.062	1.0000	-2.267	0.014	-0.88	0.67
**379**	0	1	0.000	1.0000	-2.4	0.133	0	1
**380**	-1.573	0.009	-6.123	1.0000	-2.83	0.001	-1.57	0.67
**381**	-1.03	0.073	-1.955	1.0000	-1.876	0.042	-1.03	0.67
**382**	-0.931	0.311	-1.192	0.9250	-1.564	0.141	-1.23	0.67
**383**	-1.889	0.004	-6.123	1.0000	-2.725	0.001	-1.89	0.67
**384**	-2.054	0.024	-5.283	0.9957	-2.425	0.021	-2.06	0.67
**385**	-0.709	0.103	-1.531	1.0000	-1.712	0.04	-0.71	0.67
**386**	0	1	0.000	1.0000	-0.812	0.196	0	1
**387**	0.01	0.989	-0.765	0.7901	-0.618	0.29	0.02	0.66
**388**	-0.856	0.449	-1.526	0.8599	-1.585	0.162	-0.44	0.67
**389**	-0.921	0.314	-1.191	0.9250	-1.56	0.142	-1.16	0.67
**390**	-0.611	0.164	-2.045	1.0000	-1.317	0.085	-0.61	0.67
**391**	-0.445	0.185	-1.332	1.0000	-1.158	0.082	-0.44	0.67
**392**	-1.452	0.025	-3.062	1.0000	-2.391	0.01	-1.45	0.67
**393**	-1.667	0.016	-3.062	1.0000	-2.494	0.007	-1.67	0.67
**394**	-0.95	0.04	-3.062	1.0000	-2.065	0.017	-0.95	0.67
**395**	0	1	0.000	1.0000	-1.12	0.153	0	1
**396**	0	1	0.000	1.0000	-0.812	0.196	0	1
**397**	-0.677	0.1	-1.531	1.0000	-1.905	0.038	-0.68	0.67
**398**	0	1	0.000	1.0000	-1.07	0.152	0	1
**399**	-4.174	0	-12.152	1.0000	-4.041	0	-4.18	0.67
**400**	-0.966	0.039	-3.062	1.0000	-2.082	0.016	-0.97	0.67
**401**	-0.977	0.091	-2.761	1.0000	-2.255	0.015	-0.98	0.67
**402**	0	1	0.000	1.0000	-0.764	0.188	0	1
**403**	-0.848	0.087	-1.531	1.0000	-2.071	0.029	-0.85	0.67
**404**	-0.86	0.049	-3.482	1.0000	-2.193	0.018	-0.86	0.67
**405**	0	1	0.000	1.0000	-1.117	0.155	0	1
**406**	0	1	0.000	1.0000	-1.117	0.155	0	1
**407**	-3.91	0	-9.185	1.0000	-4.442	0	-3.91	0.67
**408**	-1.761	0.018	-3.434	1.0000	-2.471	0.01	-1.76	0.67

**409**	-0.862	0.086	-1.531	1.0000	-2.111	0.025	-0.86	0.67
**410**	0	1	0.000	1.0000	-1.12	0.153	0	1
**411**	-1.604	0.012	-4.924	1.0000	-2.545	0.005	-1.6	0.67
**412**	-0.852	0.087	-1.531	1.0000	-2.105	0.025	-0.85	0.67
**413**	-2.162	0.011	-3.904	1.0000	-2.569	0.009	-2.16	0.67
**414**	-1.668	0.007	-4.592	1.0000	-2.605	0.004	-1.67	0.67
**415**	-0.821	0.101	-1.531	1.0000	-1.812	0.039	-0.82	0.67
**416**	-1.124	0.072	-2.629	1.0000	-2.268	0.015	-1.12	0.67
**417**	-0.772	0.401	-1.889	0.9131	-1.172	0.171	-0.83	0.67
**418**	-1.93	0.004	-6.123	1.0000	-2.747	0.001	-1.93	0.67
**419**	-1.281	0.089	-3.827	0.9877	-2.005	0.035	-1.56	0.67
**420**	-2.344	0.078	-3.767	0.9557	-2.578	0.052	-3.14	0.67
**421**	0	1	0.000	1.0000	-0.969	0.167	0	1
**422**	-3.496	0.001	-6.123	1.0000	-3.405	0	-3.5	0.67
**423**	-2.101	0.011	-3.910	1.0000	-2.55	0.009	-2.1	0.67
**424**	0	1	0.000	1.0000	-0.404	0.249	0	1
**425**	0.254	0.46	0.765	0.6667	-0.161	0.457	0.34	0.42
**426**	-1.777	0.018	-3.062	1.0000	-2.741	0.006	-1.78	0.67
**427**	-1.646	0.04	-5.408	0.9960	-2.419	0.011	-1.67	0.67
**428**	0	1	0.000	1.0000	-1.12	0.153	0	1
**429**	-2.184	0.005	-4.592	1.0000	-2.774	0.002	-2.18	0.67
**430**	-3.663	0	-10.845	1.0000	-5.587	0	-3.66	0.67
**431**	0	1	0.000	1.0000	-0.517	0.224	0	1
**432**	-2.549	0.002	-4.862	1.0000	-3.273	0.001	-2.55	0.67
**433**	-0.873	0.093	-1.719	1.0000	-1.794	0.044	-0.87	0.67
**434**	0	1	0.000	1.0000	-0.812	0.196	0	1
**435**	-1.516	0.017	-3.062	1.0000	-2.563	0.008	-1.52	0.67
**436**	-1.323	0.101	-4.179	0.9906	-1.999	0.037	-1.62	0.67
**437**	0	1	0.000	1.0000	-0.822	0.192	0	1
**438**	0	1	0.000	1.0000	-0.484	0.243	0	1
**439**	-1.593	0.022	-3.062	1.0000	-2.453	0.009	-1.59	0.67
**440**	-0.87	0.093	-1.718	1.0000	-1.79	0.044	-0.87	0.67
**441**	0	1	0.000	1.0000	-0.766	0.195	0	1
**442**	0	1	0.000	1.0000	-0.749	0.195	0	1
**443**	-1.166	0.237	-3.330	0.9637	-2.381	0.01	-1.71	0.67
**444**	1.791	0.138	3.791	0.2380	3.6	0.805	8.54	0.11
**445**	0	1	0.000	1.0000	-0.812	0.196	0	1
**446**	0	1	0.000	1.0000	-0.734	0.202	0	1
**447**	0.254	0.466	0.766	0.6665	-0.291	0.419	0.34	0.42
**448**	-1.958	0.005	-6.504	1.0000	-2.728	0.001	-1.96	0.67
**449**	0	1	0.000	1.0000	-0.734	0.202	0	1
**450**	0	1	0.000	1.0000	-1.117	0.155	0	1
**451**	-0.339	0.692	0.000	0.7407	-0.944	0.261	-0.89	0.67
**452**	0	1	0.000	1.0000	-0.969	0.167	0	1
**453**	-1.229	0.158	-2.649	0.9725	-1.707	0.077	-1.23	0.67
**454**	-0.847	0.081	-1.531	1.0000	-2.046	0.033	-0.85	0.67
**455**	0	1	0.000	1.0000	-0.39	0.259	0	1
**456**	-1.046	0.103	-1.952	1.0000	-1.839	0.05	-1.05	0.67
**457**	-1.063	0.069	-3.505	1.0000	-2.386	0.008	-1.06	0.67
**458**	-0.794	0.104	-1.531	1.0000	-1.784	0.041	-0.79	0.67
**459**	-1.62	0.015	-3.062	1.0000	-2.677	0.007	-1.62	0.67
**460**	-1.701	0.011	-3.968	1.0000	-2.428	0.009	-1.7	0.67
**461**	0.462	0.357	1.525	0.4685	-0.029	0.556	0.76	0.34
**462**	0.26	0.522	0.718	0.7268	-0.362	0.412	10.65	0.4
**463**	0.537	0.36	1.446	0.5265	-0.007	0.562	259.69	0.07
**464**	0	1	0.000	1.0000	-0.934	0.18	0	1

**465**	-0.699	0.35	-0.736	0.8831	-1.344	0.152	-0.95	0.67
**466**	0	1	0.000	1.0000	-0.969	0.167	0	1
**467**	-1.687	0.013	-3.062	1.0000	-2.478	0.009	-1.69	0.67
**468**	-0.425	0.177	-1.531	1.0000	-1.475	0.063	-0.42	0.67
**469**	-2.589	0.003	-4.592	1.0000	-3.245	0.001	-2.59	0.67
**470**	-2.71	0.003	-5.626	1.0000	-3.377	0.001	-2.71	0.67
**471**	-2.155	0.011	-3.793	1.0000	-2.888	0.006	-2.15	0.67
**472**	0	1	0.000	1.0000	-0.934	0.18	0	1
**473**	-0.804	0.091	-1.531	1.0000	-1.785	0.042	-0.8	0.67
**474**	-1.189	0.066	-1.913	1.0000	-2.24	0.028	-1.19	0.67
**475**	-1.182	0.065	-1.911	1.0000	-2.239	0.028	-1.18	0.67
**476**	-0.855	0.04	-3.062	1.0000	-2.241	0.015	-0.85	0.67
**477**	-3.174	0.004	-5.879	1.0000	-3.01	0.002	-3.18	0.67
**478**	-2.616	0.001	-8.891	1.0000	-3.664	0	-2.61	0.67
**479**	0	1	0.000	1.0000	-1.12	0.153	0	1
**480**	0	1	0.000	1.0000	-0.766	0.195	0	1
**481**	-0.853	0.081	-1.531	1.0000	-2.057	0.032	-0.85	0.67
**482**	0	1	0.000	1.0000	-0.825	0.19	0	1
**483**	0	1	0.000	1.0000	-0.484	0.243	0	1
**484**	0	1	0.000	1.0000	-1.562	0.1	0	1
**485**	0	1	0.000	1.0000	-1.013	0.19	0	1
**486**	-1.183	0.034	-3.301	1.0000	-2.206	0.016	-1.18	0.67
**487**	-0.614	0.224	-0.964	1.0000	-1.49	0.08	-0.61	0.67
**488**	-2.001	0.068	-3.315	0.9603	-3.588	0.022	-2.52	0.67
**489**	0	1	0.000	1.0000	-0.735	0.201	0	1
**490**	-1.758	0.025	-3.062	1.0000	-2.531	0.009	-1.76	0.67
**491**	-0.6	0.408	-0.775	0.8900	-1.3	0.149	-0.86	0.67
**492**	0	1	0.000	1.0000	-0.825	0.19	0	1
**493**	-2.452	0.033	-6.666	0.9952	-3.535	0.004	-3.01	0.67
**494**	0	1	0.000	1.0000	-0.993	0.158	0	1
**495**	-5.327	0.002	-10.785	1.0000	-4.389	0.002	-5.33	0.67
**496**	-1.183	0.066	-1.912	1.0000	-2.237	0.028	-1.18	0.67
**497**	-1.207	0.064	-1.913	1.0000	-2.251	0.027	-1.21	0.67
**498**	-1.323	0.043	-4.421	1.0000	-2.6	0.002	-1.32	0.67
**499**	-1.051	0.072	-1.952	1.0000	-1.892	0.041	-1.05	0.67
**500**	-0.611	0.111	-2.045	1.0000	-1.383	0.069	-0.61	0.67
**501**	-0.771	0.085	-1.615	1.0000	-1.997	0.033	-0.77	0.67
**502**	0	1	0.000	1.0000	-0.766	0.195	0	1
**503**	0	1	0.000	1.0000	-0.735	0.201	0	1
**504**	0	1	0.000	1.0000	-0.865	0.174	0	1
**505**	0.778	0.246	2.073	0.4028	0.584	0.729	**219.33**	**0.03**
**506**	-0.883	0.048	-3.491	1.0000	-2.213	0.017	-0.88	0.67
**507**	-2.356	0.021	-3.815	1.0000	-2.989	0.008	-2.35	0.67
**508**	-1.33	0.083	-3.827	0.9877	-2.1	0.031	-1.61	0.67
**509**	-1.671	0.011	-5.037	1.0000	-2.569	0.004	-1.67	0.67
**510**	0	1	0.000	1.0000	-1.07	0.152	0	1
**511**	0	1	0.000	1.0000	-0.822	0.192	0	1
**512**	-0.74	0.143	-1.751	1.0000	-1.646	0.051	-0.74	0.67
**513**	-1.266	0.132	-2.296	0.9630	-1.797	0.067	-1.28	0.67
**514**	-0.521	0.25	-0.956	1.0000	-1.348	0.092	-0.52	0.67
**515**	0.531	0.362	1.444	0.5268	-0.014	0.56	13.82	0.28
**516**	0	1	0.000	1.0000	-0.766	0.195	0	1
**517**	0	1	0.000	1.0000	-1.117	0.155	0	1
**518**	-1.051	0.072	-1.952	1.0000	-1.892	0.041	-1.05	0.67
**519**	0	1	0.000	1.0000	-0.993	0.158	0	1
**520**	-0.759	0.322	-0.883	0.8996	-1.424	0.143	-0.77	0.67

**521**	-0.823	0.116	-1.531	1.0000	-1.776	0.046	-0.82	0.67
**522**	-1.291	0.061	-1.910	1.0000	-2.285	0.027	-1.29	0.67
**523**	-1.626	0.017	-3.062	1.0000	-2.46	0.009	-1.63	0.67
**524**	-2.625	0.104	-4.083	0.9499	-5.243	0.002	-3.75	0.67
**525**	-0.942	0.361	-1.597	0.8951	-1.94	0.052	-1.5	0.67
**526**	-0.848	0.082	-1.531	1.0000	-2.054	0.032	-0.85	0.67
**527**	-2.402	0.009	-3.817	1.0000	-3.005	0.005	-2.4	0.67
**528**	-1.623	0.029	-4.589	1.0000	-2.73	0.001	-1.62	0.67
**529**	-0.92	0.297	-1.485	0.8827	-1.808	0.111	-1.43	0.67
**530**	0	1	0.000	1.0000	-1.562	0.1	0	1
**531**	0	1	0.000	1.0000	-1.562	0.1	0	1
**532**	-0.776	0.316	-0.877	0.8990	-1.442	0.141	-1	0.67
**533**	-0.461	0.149	-1.531	1.0000	-1.204	0.076	-0.46	0.67
**534**	-0.651	0.167	-1.841	1.0000	-1.645	0.052	-0.65	0.67
**535**	-2.091	0.015	-5.531	1.0000	-2.986	0	-2.09	0.67
**536**	-3.428	0.001	-6.123	1.0000	-4.313	0	-3.43	0.67
**537**	-1.371	0.02	-3.062	1.0000	-2.567	0.008	-1.37	0.67
**538**	-1.05	0.072	-1.952	1.0000	-1.891	0.041	-1.05	0.67
**539**	0.231	0.703	0.547	0.9336	-0.796	0.321	0.77	0.55
**540**	-4.419	0	-12.113	1.0000	-4.21	0	-4.42	0.67
**541**	0	1	0.000	1.0000	-1.562	0.1	0	1
**542**	-3.423	0.001	-6.123	1.0000	-4.301	0	-3.42	0.67
**543**	-1.612	0.045	-2.869	1.0000	-2.597	0.006	-1.61	0.67
**544**	-2.515	0.019	-3.823	1.0000	-3.071	0.008	-2.51	0.67
**545**	0	1	0.000	1.0000	-1.117	0.155	0	1
**546**	-0.802	0.118	-1.531	1.0000	-1.752	0.047	-0.8	0.67
**547**	-1.381	0.056	-2.840	1.0000	-2.503	0.008	-1.38	0.67
**548**	0	1		1.0000	-2.4	0.133	0	1
**549**	-6.184	0	-9.499	1.0000	-10.84	0	-6.18	0.67
**550**	-1.018	0.083	-2.637	1.0000	-2.196	0.017	-1.02	0.67
**551**	0	1	0.000	1.0000	-0.764	0.188	0	1
**552**	0	1	0.000	1.0000	-0.934	0.18	0	1
**553**	0	1	0.000	1.0000	-1.117	0.155	0	1
**554**	0	1	0.000	1.0000	-1.562	0.1	0	1
**555**	-0.847	0.087	-1.531	1.0000	-2.073	0.029	-0.85	0.67
**556**	-1.629	0.016	-3.983	1.0000	-2.379	0.012	-1.63	0.67
**557**	-2.908	0.002	-4.902	1.0000	-3.509	0.001	-2.91	0.67
**558**	-0.95	0.077	-1.531	1.0000	-2.094	0.028	-0.95	0.67
**559**	0	1	0.000	1.0000	-0.969	0.167	0	1
**560**	-2.612	0.001	-8.151	1.0000	-2.875	0.001	-2.61	0.67
**561**	-1.743	0.024	-4.379	1.0000	-2.703	0.001	-1.74	0.67
**562**	-1.347	0.023	-3.062	1.0000	-2.561	0.007	-1.35	0.67
**563**	0.287	0.436	0.765	0.6668	-0.123	0.477	0.58	0.4
**564**	0	1	0.000	1.0000	-1.117	0.155	0	1
**565**	-0.842	0.319	-3.113	0.9563	-1.47	0.099	-1.22	0.67
**566**	-2.823	0.003	-6.123	1.0000	-3.086	0.001	-2.82	0.67
**567**	-0.95	0.077	-1.531	1.0000	-2.124	0.024	-0.95	0.67
**568**	-0.819	0.081	-1.531	1.0000	-1.786	0.044	-0.82	0.67
**569**	-0.612	0.13	-2.045	1.0000	-1.365	0.073	-0.61	0.67
**570**	0	1	0.000	1.0000	-2.4	0.133	0	1
**571**	0.47	0.564	1.108	0.8478	-0.452	0.461	0.47	0.48
**572**	0	1	0.000	1.0000	-0.766	0.195	0	1
**573**	0	1	0.000	1.0000	-0.484	0.243	0	1
**574**	-1.749	0.223	-3.176	0.9216	-1.58	0.114	-3.01	0.67
**575**	0	1	0.000	1.0000	-0.487	0.241	0	1
**576**	-0.868	0.094	-1.718	1.0000	-1.789	0.045	-0.87	0.67

**577**	0	1	0.000	1.0000	-0.819	0.193	0	1
**578**	0	1	0.000	1.0000	-0.822	0.192	0	1
**579**	0	1	0.000	1.0000	-1.013	0.19	0	1
**580**	-0.584	0.513	-0.578	0.8317	-0.716	0.295	-1.11	0.67
**581**	0	1	0.000	1.0000	-0.819	0.193	0	1
**582**	-1.592	0.039	-3.721	1.0000	-2.613	0.012	-1.59	0.67
**583**	-1.055	0.157	-3.392	0.9843	-1.42	0.101	-1.06	0.67
**584**	-1.025	0.101	-1.954	1.0000	-1.86	0.044	-1.03	0.67
**585**	-1.814	0.017	-3.438	1.0000	-2.491	0.009	-1.81	0.67
**586**	-1.883	0.012	-6.134	1.0000	-2.586	0.004	-1.88	0.67
**587**	-1.029	0.037	-3.549	1.0000	-2.337	0.013	-1.03	0.67
**588**	-1.893	0.012	-6.134	1.0000	-2.59	0.004	-1.89	0.67
**589**	-3.6	0.001	-7.947	1.0000	-3.293	0	-3.6	0.67
**590**	0	1	0.000	1.0000	-1.117	0.155	0	1
**591**	0	1	0.000	1.0000	-0.767	0.187	0	1
**592**	-1.414	0.021	-3.062	1.0000	-2.592	0.007	-1.41	0.67
**593**	0.254	0.46	0.765	0.6667	-0.198	0.436	0.4	0.41
**594**	-0.825	0.1	-1.531	1.0000	-1.776	0.046	-0.82	0.67
**595**	0.254	0.465	0.766	0.6666	-0.335	0.396	0.34	0.42
**596**	-1.417	0.012	-4.592	1.0000	-2.494	0.005	-1.42	0.67
**597**	0	1	0.000	1.0000	-0.766	0.195	0	1
**598**	-0.747	0.245	-2.296	0.9630	-1.321	0.098	-1.01	0.67
**599**	0	1	0.000	1.0000	-0.802	0.172	0	1
**600**	0.289	0.435	0.765	0.6667	-0.169	0.453	0.56	0.4
**601**	0	1	0.000	1.0000	-0.484	0.243	0	1
**602**	-0.858	0.086	-1.531	1.0000	-2.079	0.029	-0.86	0.67
**603**	-1.193	0.065	-1.913	1.0000	-2.244	0.027	-1.19	0.67
**604**	0.281	0.456	0.725	0.7036	-0.145	0.468	323.71	0.15
**605**	0	1	0.000	1.0000	-0.822	0.192	0	1
**606**	-0.239	0.383	-0.876	1.0000	-0.499	0.183	-0.24	0.67
**607**	0.835	0.267	2.106	0.4160	0.362	0.629	40.23	0.19
**608**	0	1	0.000	1.0000	-0.934	0.18	0	1
**609**	-0.801	0.341	-1.261	0.9316	-1.049	0.19	-1.1	0.67
**610**	-0.311	0.708	0.000	0.7407	-0.678	0.291	-0.66	0.67
**611**	-2.079	0.02	-3.930	1.0000	-2.538	0.009	-2.08	0.67
**612**	-2.544	0.005	-8.177	1.0000	-2.846	0.001	-2.54	0.67
**613**	0	1	0.000	1.0000	-2.4	0.133	0	1
**614**	0	1	0.000	1.0000	-0.969	0.167	0	1
**615**	0.204	0.462	0.681	0.7489	-0.064	0.491	0.2	0.42
**616**	-1.877	0.004	-6.135	1.0000	-2.602	0.003	-1.88	0.67
**617**	-1.397	0.045	-3.703	1.0000	-2.603	0.003	-1.4	0.67
**618**	0.227	0.37	0.765	0.6669	0.044	0.545	0.23	0.36
**619**	-1.844	0.036	-2.886	1.0000	-2.677	0.005	-1.84	0.67
**620**	0.198	0.847	1.531	0.4609	-0.051	0.465	0.31	0.62
**621**	0	1	0.000	1.0000	-1.562	0.1	0	1
**622**	-0.834	0.088	-1.531	1.0000	-1.817	0.04	-0.83	0.67
**623**	-0.567	0.42	-0.765	0.8889	-0.958	0.189	-0.83	0.67
**624**	0.818	0.188	2.296	0.2963	0.517	0.73	32.26	0.13
**625**	0	1	0.000	1.0000	-0.766	0.195	0	1
**626**	0.405	0.373	0.875	0.6362	-0.225	0.45	57.82	0.28
**627**	-0.644	0.168	-1.840	1.0000	-1.629	0.053	-0.64	0.67
**628**	0	1	0.000	1.0000	-0.993	0.158	0	1
**629**	0	1	0.000	1.0000	-0.764	0.188	0	1
**630**	0	1	0.000	1.0000	-0.204	0.346	0	1
**631**	0.585	0.13	1.531	0.4444	0.304	0.682	1.44	0.15
**632**	0	1	0.000	1.0000	-0.391	0.261	0	1

**633**	-0.819	0.116	-1.531	1.0000	-1.771	0.046	-0.82	0.67
**634**	0	1	0.000	1.0000	-0.479	0.24	0	1
**635**	0	1	0.000	1.0000	-0.391	0.261	0	1
**636**	0	1	0.000	1.0000	-0.402	0.251	0	1
**637**	0	1	0.000	1.0000	-0.391	0.261	0	1
**638**	0	1	0.000	1.0000	-0.47	0.251	0	1
**639**	-1.849	0.013	-3.114	1.0000	-2.779	0.006	-1.85	0.67
**640**	-0.308	0.71	0.000	0.7407	-0.614	0.313	-0.85	0.67
**641**	-1.211	0.064	-1.912	1.0000	-2.253	0.027	-1.21	0.67

p values < 0.05 or posterior probability > 0.9 are shown in bold.
